# Emerging roles of exosomes in oral diseases progression

**DOI:** 10.1038/s41368-023-00274-9

**Published:** 2024-01-15

**Authors:** Jiayi Wang, Junjun Jing, Chenchen Zhou, Yi Fan

**Affiliations:** 1 State Key Laboratory of Oral Diseases & National Center for Stomatology & National Clinical Research Center for Oral Diseases & Department of Cariology and Endodontics, West China Hospital of Stomatology, Sichuan University, Chengdu, China; 2 State Key Laboratory of Oral Diseases & National Center for Stomatology & National Clinical Research Center for Oral Diseases & West China Hospital of Stomatology, Sichuan University, Chengdu, China; 3 State Key Laboratory of Oral Diseases & National Center for Stomatology & National Clinical Research Center for Oral Diseases & Department of Pediatric Dentistry, West China Hospital of Stomatology, Sichuan University, Chengdu, China

**Keywords:** Periodontitis, Oral cancer detection

## Abstract

Oral diseases, such as periodontitis, salivary gland diseases, and oral cancers, significantly challenge health conditions due to their detrimental effects on patient’s digestive functions, pronunciation, and esthetic demands. Delayed diagnosis and non-targeted treatment profoundly influence patients’ prognosis and quality of life. The exploration of innovative approaches for early detection and precise treatment represents a promising frontier in oral medicine. Exosomes, which are characterized as nanometer-sized extracellular vesicles, are secreted by virtually all types of cells. As the research continues, the complex roles of these intracellular-derived extracellular vesicles in biological processes have gradually unfolded. Exosomes have attracted attention as valuable diagnostic and therapeutic tools for their ability to transfer abundant biological cargos and their intricate involvement in multiple cellular functions. In this review, we provide an overview of the recent applications of exosomes within the field of oral diseases, focusing on inflammation-related bone diseases and oral squamous cell carcinomas. We characterize the exosome alterations and demonstrate their potential applications as biomarkers for early diagnosis, highlighting their roles as indicators in multiple oral diseases. We also summarize the promising applications of exosomes in targeted therapy and proposed future directions for the use of exosomes in clinical treatment.

## Introduction

The oral cavity is the initial segment of the digestive system, second source of respiration, and a crucial organ for pronunciation, mastication, and facial esthetics. Poor oral health may have an impact on a person’s overall health, causing pain, discomfort, and disfigurement.^1^ Oral diseases, such as dental caries, periodontal diseases, and oral cancers, affect nearly 3.5 billion people, which are global burdens that cause patients suffering, especially those with a low socioeconomic status.^2^ To alleviate these burdens, researchers have come up with innovative methods for early diagnosis and effective treatment.

Extracellular vesicles (EVs) are derived from cellular membranes and released into the extracellular space; they play critical roles in intercellular communication.^3^ There are two main categories of EVs, including exosomes and ectosomes.^4^ Exosomes, which are principal constituents of EVs, are derived from the endosomal system and possess bilayer lipid encapsulation, with diameters ranging from 30 to 150 nm.^5^ Compared with ectosomes, which assemble cargos on the cytosolic surface and transient release in “outward budding”,^6^ exosomes exhibit more intricate interactions with cyto-inclusions and have garnered significant attention from biological researchers worldwide for their abundant cargos and various functions associated with physiological or pathological processes.^5^

The biogenesis of exosomes is tightly regulated through a complex network of processes. Upon endocytosis, the potential cargos are internalized by the cells and give rise to early-sorting endosomes (ESEs). The subsequent interactions with organelles, such as the endoplasmic reticulum (ER) and Golgi apparatus, lead to the maturation of ESEs into late-sorting endosomes (LSEs). Following this, the cargos accumulate near the limiting membrane of multivesicular bodies (MVBs) and generate intraluminal vesicles (ILVs), which will eventually be released as exosomes via exocytosis.^5,7,8^ It should be noted that some ILVs may also undergo interactions with lysosomes or autophagosomes.^5,7,8^ Throughout the entire process, various regulators, such as the endosomal sorting complex required for transport (ESCRT), tetraspanin CD9/CD63/CD81, and Alix/programmed cell death 6-interacting protein (PDCD6IP), are involved in the intricate mechanisms of sorting and secretion.^5,7–9^ Accordingly, an extensive body of research has confirmed that the final version of the exosomes contains diversified contents, including amino acids, proteins, nucleic acids, and cellular metabolites. These molecules play distinct roles in intercellular signaling transmission, immune-modulation, stromal adaptation, and multiple biological events.^9^

Therefore, exosomes exhibit significant potential in managing disease processes. Previous research has elucidated the close correlation between exosomes and oral diseases, including periodontal inflammation, oral squamous cell carcinomas (OSCCs), oral mucosa diseases, etc. The application of exosomes involves monitoring the progression of the diseases, early diagnosis, detection through specific manifestations in fluid, and advanced targeted therapy via precise molecule delivery. This review concludes the recent studies on the applications of exosomes in oral diseases and aims to provide a comprehensive insight into the latest developments in exosome alterations, functions, and applications in oral-related physiological and pathological conditions. The ultimate goal is to identify new opportunities for the effective utilization of exosomes in the prevention and treatment of oral diseases.

## Exosomes in the progression of oral diseases

As researchers have demonstrated the intricate roles of exosomes in biological events, their distinct alterations are significant components in the pathological processes of oral diseases. At the initial stage, analyzing the exosomal information helps to improve our early awareness of oral diseases. During the development and prognosis stages, summarizing the exosome-related manifestations can enhance our comprehension of the selection and response of treatments. Therefore, exosomes serve as a crucial indicator in the diagnosis, monitoring, and treatment of several oral diseases. In this part, we summarize the roles of exosomes in determining the progression of oral diseases, with a focus on periodontal diseases and oral cancers.

### Periodontal and bone-related pathological status

#### Periodontal inflammation and bone resorption

Following exposure to risk factors (intrinsic and/or acquired), the pathological changes in periodontitis are initiated by immune-inflammatory responses, leading to the release of inflammatory molecules, such as cytokines and matrix metalloproteinases (MMPs). These molecules exert their effects on periodontal tissues, thereby inducing clinical manifestations.^10^ Osteoclasts are activated in inflammatory microenvironments and lead to the destruction of the surrounding alveolar bone. Subsequently, these irreversible destructions of periodontal structures accelerate the progression of periodontitis and lead to tooth mobility or even tooth loss, severely impacting the patients’ quality of life.^11^ The previous studies have demonstrated the significance of inflammatory factors in periodontitis.^10^ It is noteworthy that exosomes play important roles in the modulation and alteration of periodontal inflammation and bone resorption.^12^

Periodontal ligament stem cell (PDLSC)-derived exosomes significantly improved angiogenesis in inflammatory regions by upregulating the vascular endothelial growth factor (VEGF) in human umbilical vein endothelial cells (HUVECs) via miR-17-5p.^13^ Salivary exosomal miR-223-3p increased the interleukin (IL)-1β and IL-6 levels by mediating NLRP3 gene expression and pyroptosis.^14^ Oxidative stress is involved in periodontitis progression with abnormal reactive oxygen species (ROS). Protein arginine methyltransferase 1 (PRMT1) induced by oxidative stress inhibited exosome secretion from periodontal ligament cells (PDLCs), resulting in reduced osteogenic differentiation.^15^

The interactions between the host inflammatory response and microbe are also closely involved in the progression of periodontitis.^10^ Similar to exosomal microRNAs (miRNAs) derived from host cells, small RNAs of miRNA size (msRNAs) from pathogens (*A. actinomycetemcomitans, P. gingivalis, and T. denticola*) were detected in the bacterial outer membrane vesicles.^16^ *P. gingivalis* can also assign a senescence-associated secretory phenotype (SASP) to dendritic cells (DCs) and T cells by exosomes, and ultimately, this results in alveolar bone loss.^17^ Moreover, mechanical stimuli are a promoter of periodontal inflammatory and tissue/bone impairment, mainly acting on PDLCs.^18^ PDLCs secrete exosomal miR-9-5p when facing cyclic stretching and promote the M1 (pro-inflammatory) polarization of macrophages through the miR-9-5p/SIRT1/NF-kB pathway in murine models.^19^ The M1 macrophage was also induced by periodontal ligament fibroblast-derived exosomes when a compressive force was applied. The underlying mechanism was associated with the Yes-associated protein (YAP),^20^ which is a crucial component in the Hippo signaling pathway and performs significant roles in cellular mechanotransduction.^21^ In the immune-modulation of the inflammatory response, the YAP/Hippo pathway acts as a key upstream regulator.^22^ These studies highlight the complex interactions between exosomes and the clinical manifestations of periodontal diseases, according to which we should take more steps to attenuate the progression of periodontitis.

#### Orthodontic movement

Orthodontic treatment involves the complex processes of alveolar bone remodeling upon the application of mechanical forces. During movement, the expression of gingival crevicular fluid (GCF) exosomal miR-29 is significantly increased.^23^ Meanwhile, the PDLSC-derived exosomal miRNAs are largely altered,^24,25^ and the quantity of exosomal proteins annexin A3 (ANXA3) increases, which induces osteoclast differentiation by the activation of extracellular regulated protein kinase (ERK).^26^ When orthodontic movement stops and teeth position satisfies our needs, PDLSC-derived exosomes also contribute to teeth stabilization. For instance, Simvastatin, a bone-formation-enhancing drug, has better bioavailability in conjunction with PDLSC-derived exosomes.^27^

### Oral squamous cell carcinomas

**Fig. 1:**
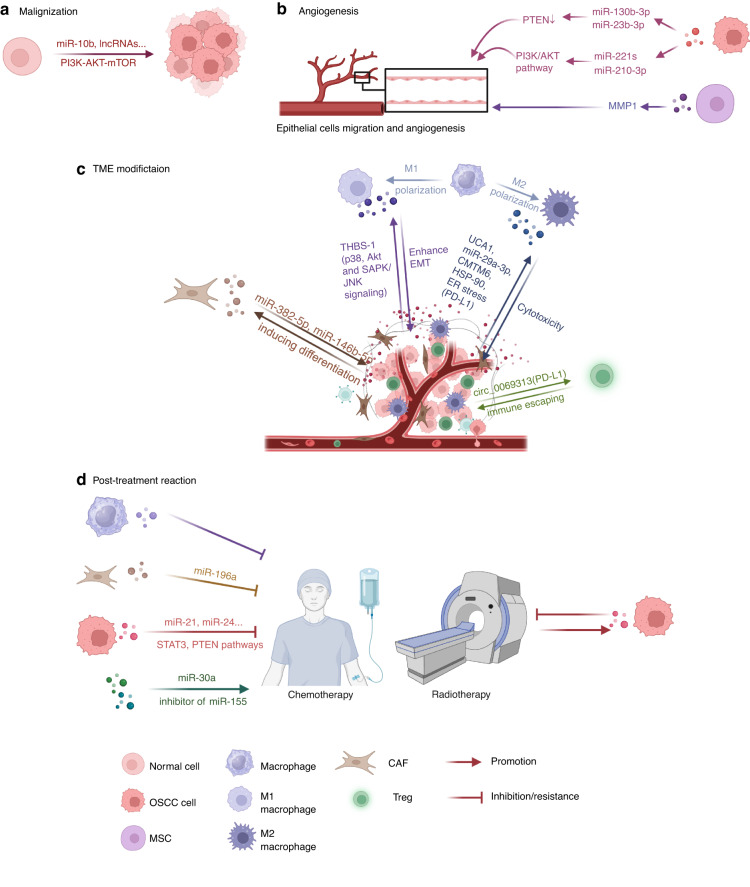
Exosomes in OSCCs. Exosomes play crucial roles in several key processes through OSCCs progression. **a** Addition of exosomal miR-10b and increased level of several exosomal lncRNAs (targeting PI3K-AKT-mTOR pathway) lead to initiation of OSCCs. **b** OSCC cells derived exosomal miR-130b-3p and miR-23b-3p promoted angiogenesis through downregulating PTEN, while miR-221s and miR-210-3p activated through PI3K/AKT pathway. MSCs-derived MMP1 played the same role in angiogenesis. **c** During the progression of OSCCs, TME exhibits intricate interactions with tumors. Exosomes derived from OSCC cells promoted differentiation of CAFs, while CAFs secreted exosomal miR-382-5p and miR-146b-5p to enhance tumor development. In immune regulations, cancer-derived exosomal THBS-1 induced polarization of TAMs toward M1 type. Exosomal UCA1, miR-29a-3p, CMTM6, HSP-90 and PD-L1 (under ER stress) lead TAMs to M2 type. Both M1 and M2 macrophages contribute to the progression of malignancy. T cells were also modulated by cancer cells through exosomal circ_0069313, targeting PD-L1. In addition, Tregs played a crucial role in facilitating immune evasion in OSCCs. **d** Exosomes can regulate treatment response of OSCC to chemotherapy and radiotherapy

With increasing incidence and mortality rates, oral cancers rank as the 13th most common cancer worldwide, with an estimated 377,713 new cases and 177,757 deaths in 2020.^28,29^ The consumption of tobacco and areca nut significantly increases the risk of oral cancers. Specifically, oral cancers cause larger burdens in developing countries due to delayed diagnosis and limited treatment opportunities.^29^ Among all the types of oral cancers, OSCCs are the most common, with an estimated 40% higher incidence rate in 2040.^30^ Summarizing the alterations in the exosomes in OSCCs could enhance our understanding of malignant progression. Here, we discuss exosomal roles in malignization, angiogenesis, and tumor microenvironment (TME) modification in OSCC progression and the post-treatment response (Fig. 1).

#### Malignization

According to transcriptome analysis, head and neck squamous cell carcinoma (HNSCC)-associated exosomes play a significant role in various processes throughout cancer development.^31^ As the most common malignancy types in HNSCCs, OSCCs exhibit various exosomal alterations. From the outset, the injection of OSCC-tumor-derived exosomes accelerated the malignancy progression of precancerous lesions in murine models,^32^ and this phenomenon was attributed to exosomal miR-10b through AKT signaling.^33^ The same initiation of malignancy also occurs in recurrent OSCCs with increased serum exosomal long non-coding RNA (lncRNA)-CCDC144NL-AS1 and MAGI2-AS3 via the PI3K-AKT-mTOR pathway^34^ (Fig. 1a). Upon the manifestation of these early malignancy symptoms, the subsequent progression of OSCCs ensues through diverse exosomal alterations.

#### Angiogenesis

Inducing the vasculature is an important hallmark of cancer,^35^ and the exosomes accelerate this process through several pathways (Fig. 1b). Phosphatase and tensin homolog deleted on chromosome ten (PTEN) is a tumor-suppressing factor, and its expression is downregulated in OSCCs.^36^ Exosomal miR-130b-3p derived from OSCCs cells and miR-23b-3p from salivary adenoid cystic carcinomas (SACCs) cells negatively regulate PTEN expression, promoting migration and angiogenesis in HUVECs.^37,38^ miR-221s and miR-210-3p also regulated HUVECs angiogenesis through the PI3K/AKT pathway.^39,40^ Besides cancer cells, the mesenchymal stem cells (MSCs) in oral carcinomas can secrete angiogenesis-stimulative exosomes. OSCC–MSC-derived exosomal matrix metalloproteinases 1 (MMP1) significantly enhances the function of HUVECs.^41^

#### TME modification

In addition to tumor cells, TME exerts a dominant influence on the development of OSCCs, involving intricate interactions with exosomes (Fig. 1c). The TME is composed of the extracellular matrix (ECM) and relevant cells, including cancer-associated fibroblasts (CAFs), endothelial cells, and immune cells.^42^ In OSCCs, tumor-derived exosomes induce CAF differentiation.^43^ In turn, the CAFs have received great attention for their significance in promoting tumor development through intracellular communications, primarily via exosome secretion.^44^ In OSCCs, the CAF-derived miR-382-5p^45^ and miR-146b-5p^46^ exosomes are upregulated, leading to the invasion of cancer cells and metastasis. In addition, the miR-34a-5p-devoid exosomes from CAFs promote malignancy by targeting the AXL (a component of the receptor tyrosine kinase) of cancer cells.^47^

By analyzing the genes related to EV formation in the HNSCCs samples and their impact on cellular behaviors, other scientists have found a significant correlation between EVs and immune modification (T/B cells, macrophage, and neutrophils) when a malignancy occurs.^48^ The tumor-associated macrophages (TAMs) play a crucial role as immune cells within the TME. Generally, the TAMs mainly polarize into two states—the M1 and M2 subtypes.^49^ According to the conventional perspective, the M1 macrophages perform anti-tumor functions by direct cytotoxicity or antibody-dependent cell-mediated cytotoxicity (ADCC), while the M2 macrophages promote cancer progression and metastasis by secreting cytokines and related molecules, such as ILs, epithelial growth factors (EGFs), and MMPs.^50^ However, the involvement of M1 macrophages in the process of malignancy invasion has been recently observed, suggesting that their presence may contribute to more aggressive malignization, but not a higher survival rate.^51^

In OSCCs, the TAMs have complex interactions with the tumor cells. The exosomal transforming growth factor beta (TGF-β) derived from HNSCCs cells can promote angiogenesis by both interacting with epithelial cells and modulating TAMs chemotaxis for pro-angiogenic functions.^52^ OSCC-derived exosomal thrombospondin 1 (THBS-1) activates an M1-like macrophage through p38, Akt, and SAPK/JNK signaling, which promotes cancer progression.^53^ M1-like TAMs enhance the OSCC epithelial–mesenchymal transition (EMT) and cancer stem cell formation through the IL-6/Jak/signal transducer and the activator of transcription 3 (Stat3)/THBS-1 axis.^54^

Furthermore, oral cancer cells are closely associated with the conventional oncogenic M2 macrophages through multiple pathways.^50^ OSCC cancer stem cell-derived exosomes polarize TAMs into M2 macrophages by the urothelial carcinoma-associated 1 (UCA1) secretion targeting LAMC2-PI3K/AKT signaling pathway. These exosomes also suppress anti-tumor immunity, including CD4^+^ T cells activation and interferon-γ (IFN-γ) production.^55^ In addition, OSCC cancer cells secrete exosome-enclosed cargos, inducing M2 macrophages. MiR-29a-3p, a member of the miR-29 family that significantly increases in OSCCs,^56^ exerts effects on M2 polarization through the suppressor of cytokine signaling 1 (SOCS1)/signal transduction and the transcriptional activator (STAT) pathway.^57^ The CKLF-like MARVEL transmembrane domain-containing 6 (CMTM6)^58^ and heat shock protein-90 (HSP-90)^59^ are important proteinic influencers of M2 macrophage conversion, indicating novel crosstalk between cancer cells and immune-modulation. ER stress, a functional proteinaceous response that reacts to cellular events like oncogenesis, drives OSCC cells to secrete exosomal programmed death ligand 1 (PD-L1) and activate the M2 polarization of TAMs.^60^

Besides TAMs, T cells are also important immune regulators in OSCCs. T-effector (Teff) cells and T-regulatory (Treg) cells are the two main classifications of T cells.^61^ Teff cells primarily execute killing functions toward pathogens or, anomaly, self-antigens. Treg cells modulate over-functioning Teff cells and maintain immune homeostasis. However, in the oncogenesis process, Treg cells may lead to immune evasion.^61^ In OSCCs, exosomal circular RNA circ_0069313 could increase the PD-L1 expression in cancer cells with miR-325-3p sponging and interact with Treg cells. Thus, the Teff cells are suppressed, while the Treg cells are activated, resulting in the immune escaping of malignancy.^62^

#### Post-treatment reaction

The traditional non-surgical treatments for OSCCs include chemotherapy and radiotherapy.^63^ However, resistance and unwilling treatment responses will impact the prognosis and mortality. Therefore, it is imperative to identify precise markers that can effectively assess the efficacy of these therapies. Chemoresistance occurs in nearly all anti-tumor drugs, and the underlying mechanism can be intrinsic (gene mutations) or acquired (TME, epigenetic alteration).^64^ According to recent studies, the exosomes play a crucial role in chemoresistance of OSCCs through several pathways^65^ (Fig. 1d). Exosomal microRNAs (such as miR-21 and miR-24) derived from OSCC cell lines contribute to chemoresistance by targeting multiple signaling molecules, including STAT3 and PTEN.^65,66^ Macrophage-derived exosomes and CAF-derived exosomal miR-196a can also reduce drug sensitivity.^67,68^ In addition, the exosomes can influence chemoresistance by modulating the drug efflux, vesicular pH, anti-apoptotic signaling, DNA damage repair (DDR), as well as the EMT.^65^ In reverse, the delivery of exosomal miR-30a and the inhibitor of exosomal miR-155 showed the ability to enhance the sensitivity of cisplatin-resistant OSCCs,^69,70^ highlighting their potential role in improving cancer therapy. Cancer cells’ reactions toward radiotherapy are also associated with exosomes (Fig. 1d), mainly through modulating DDR, cell death signals, and the EMT.^71^ In HNSCC radiation-resistance cases, the functions of tumor-promoting exosomes are strengthened after radiation.^72^

Besides resistance, the profile of exosomes in OSCCs alters in response to treatment. After surgery and/or chemo-radiotherapies, monitoring immune-related exosomal proteins (such as PD-L1) could accurately indicate the treatment reaction and recurrence possibility.^73,74^ Melatonin, a hormone secreted by the pineal gland, plays crucial roles in multiple physiological processes.^75^ The recent research has highlighted its potential function in OSCC therapy as an adjuvant, owing to its ability to inhibit tumor progression by modulating key regulators, such as MMP-9, p53, and epidermal growth factor receptor (EGFR), as well as enhancing immune functions.^76,77^ The expression of OSCC cell-derived exosomes (miR-21 and miR-155) undergoes alterations following melatonin application, indicating their potential for assessing the treatment response and predicting the prognosis.^78^

In conclusion, exosomes exhibit various characteristics during the development and prognosis of OSCCs. By improving our comprehension of these alterations, we can approach a better understanding of the enigma surrounding OSCC occurrence and conduct further investigations on therapeutic interventions.

## Early diagnostic methods utilizing exosomes in oral diseases

According to the above, it is convincing that exosomes play a crucial role in the progression of oral diseases. Our particular emphasis lies in exosomal application for early diagnosis due to the potential exacerbation of patients’ suffering caused by delayed detection. In contrast to the more apparent biomarkers, such as inflammatory molecules of periodontitis and gene expression changes in OSCCs, exploring the full advantages of exosomes represents a promising avenue. Liquid biopsy is an emerging disease diagnostic method that primarily involves the isolation and evaluation of fluid entities, such as DNAs/RNAs, proteins, and extracellular vesicles in human saliva, blood, and urine.^79^ Compared with the other patterns of biomarkers, exosomes protect their cargos, remarkably enhancing the accuracy and practicability of detection.^80^ In the field of oral medicine, exosome-associated liquid biopsy has demonstrated value in the diagnosis of multiple diseases.^81^ The identification of novel biomarkers and reliable detective techniques are potential research prospects.

### Biomarkers for periodontitis

The traditional diagnostic criteria for periodontitis primarily rely on clinical symptoms, such as the periodontal pocket depth and the pathological loss of the alveolar bone. However, the possibilities of “overdiagnosis” or “underdiagnosis” remain unsolved, as these clinical variables may fail to accurately predict the disease progression and treatment response.^82^ The identification of exosomal biomarkers has the potential to enhance our understanding of the intricate biological mechanisms underlying periodontitis progression and facilitate the development of advanced clinical management strategies for patients. Generally, the concentration of EVs from the GCF of periodontitis patients is evidently higher when compared to that of healthy samples.^83^

Nucleic acids, as prominent constituents of exosomal cargos, can provide valuable diagnostic insights. In addition to DNA/RNAs that directly encode proteins, non-coding RNAs play important roles in regulating cellular events through diverse pathways, such as gene silencing and post-translational modification.^84^ MiRNAs are small, non-coding RNAs that modulate mRNA expression by forming RNA-induced silencing complexes (RISCs) or directly binding to mRNAs through base pairing.^85^

According to Kamal et al.,^86^ 1995 salivary exosomal miRNAs and 333 plasma exosomes were significantly altered in their periodontitis samples. Among these potential biomarkers, serum exosomal miR-let-7d, miR-126-3, miR-199a-3, and salivary exosomal miR-125a-3 were notable for distinguishing the disease status and correlation with the clinical stages.^86^ In addition, researchers reported that serum exosomal miR-1304-3p, miR-200c-3p, small nucleolar RNA SNORD57, SNODB1771,^87^ salivary exosomal miR-223-3p,^14^ Osx mRNA^88^ and GCF exosomal miR-1226^89^ were downregulated in periodontitis. However, salivary exosomal miR-140-5p, miR-146a-5,miR-628-5p,^90^ miR-381-3p,^91^ tumor necrosis factor-alpha (TNF-α),^88^ and PD-1 mRNAs exhibited higher expression levels in the periodontitis samples.^92^ As for proteins, the levels of the tetraspanins CD9 and CD81 were decreased in the salivary exosomes of periodontitis, which is associated with an inflammatory reaction.^93^ The levels of immune-related proteins, such as complement components (C6, C8A, and C8B) and chemokines, were increased in the salivary exosomes of young severe periodontitis patients,^94^ implying that immune changes are responsible for this pathological alteration.

### Biomarkers for oral cancers

Scalpel biopsies and histological examinations have long been regarded as the golden standard for malignancy research.^95^ However, oral cancers, particularly OSCCs, usually develop imperceptibly, with minimal clinical manifestations in the early stages. Therefore, by the time the symptoms have been identified, the malignancy may have already appeared, leading to a poor prognosis.^96^ In the meantime, the pursuit of non-invasive detection methods has become a prevailing trend in the medical field. These statuses emphasize the necessity for the application of exosomal biomarkers in OSCC diagnosis. Compared with the samples from healthy controls, the exosomes from OSCC patients showed an increased concentration and larger diameter, with higher CD63 and lower CD9/CD81 expression levels, revealing basic evidence for distinguishment.^97^ However, the most comprehensive and convincible distinctions are the cargos of the exosomes. We have summarized these biomarkers and the relevant research in Table 1.

**Table 1:** Exosomal cargos as biomarkers for oral cancer diagnosis

Tissue	Sample	Exo-biomarker	Alternation	Statistic scale	Mechanism	Other information	Ref
Tongue SCCs	Plasma	miR-19a/27b/20a/28-3p/200c/151-3p/223/20b	Upregulated	5 patients	N/A	N/A	^98^
		miR-370/139-5p/let-7e/30c	Downregulated				
OSCCs	Plasma	miR-130a	Upregulated	184 patients 196 controls	N/A	Associated with poor prognosis (advanced TNM stages/poorly differentiation)	^99^
OSCCs	Serum	miR-155/21	Upregulated	35 patients 11 controls	Downregulating tumor suppressors PTEN and Bcl-6	N/A	^100^
		miR-126	Downregulated		Inhibiting oncogene EGFL7 expression	Low miR-126 was associated with poor prognosis	
HNSCCs	Serum	miR-3168/125a-5p/451a/16-2-3p	Upregulated	22 patients 10 controls (with benign neoplasm)	N/A	N/A	^101^
OOCs	Saliva	miR-486-5p	Upregulated	25 patients 25 controls	N/A	Higher level in stage II	^105,106^
		miR-10b-5p	Downregulated			N/A	
OSCCs	Saliva	miR-24-3p	Upregulated	49 patients 14 controls	Targeting circadian gene-PER1	N/A	^102^
OSCCs	Saliva	miR-1307-5p	Upregulated	12 patients 7 controls	Suppressing onco-related genes THOP1, EHF, RNF4, GET4 and RNF114	Associated with poor prognosis	^103^
OSCCs	Saliva	miR-134/200a IL-1β and IL-8	Upregulated	14 patients 23 controls	N/A	N/A	^104^
OSCCs	Serum	circ_0000199	Upregulated	108 patients 50 controls	Associated with miR-145-5p and miR29b-3p	Associated with poor prognosis	^110^
HNSCCs	Plasma	TGF-β	Upregulated	36 patients 12 controls	TME-dependent oncogenic role	Increasing with stages/size of tumors	^111^
OSCCs	Serum/saliva	Alix	Upregulated	Serum 29 patients 21 controls Saliva 23 patients 20 controls	N/A	Only serum exoAlix associated with stages	^112^
OSCCs	Serum	Combined CRP, VWF and LRG	Upregulated	40 patients 20 controls	N/A	Combined biomarkers show greater sensitivity and specificity than individual ones.	^113^
OSCCs	Serum	PF4V1, CXCL7, F13A1 and ApoA1	Downregulated (F13A1 upregulated)	10 patients (no LNM) 10 patients (with LNM) 10 controls	N/A	PF4V-tumor differentiation level PF4V1/F13A1-positive node number ApoA1-smoking and drinking	^114^

*SCC* squamous cell carcinoma, *OSCC* oral squamous cell carcinoma, *HNSCC* head and neck squamous cell carcinoma, *OOC* oral and oropharyngeal cancer, *miR* micro-RNA, *IL* interleukin, *circRNA* circular RNA, *TGF-β* transforming growth factor beta, *Alix* programmed cell death 6-interacting protein (PDCD6IP), *CRP* C-reactive protein, *VWF* von Willebrand factor, *LRG* leucine-rich alpha-2-glycoprotein, *PF4V1* platelet factor 4 variant, *CXCL7* C-X-C motif chemokine, *F13A1* coagulation factor XIII, A, *ApoA1* apolipoprotein A-I, *PTEN* chromosome ten, *PER1* Period1

In OSCCs, the altered secretion of exosomal miRNAs can signal the disease status. Based on the statistical evidence, the levels of serum/plasma exosomal miR-19a/27b/20a/28-3p/200c/151-3p/223/20b,^98^ miR-130a,^99^ miR-155, miR-21,^100^ miR-3168, miR-125a-5p, miR-451a, and miR-16-2-3p,^101^ and salivary exosomal miR-486-5p, miR-486-3p, miR-24-3p,^102^ miR-1307-5p,^103^ miR-200a, and miR-134^104^ were significantly elevated in the patients with OSCCs, performing potential oncogenic roles. However, the serum exosomal miR-370/139-5p/let-7e/30c,^98^ miR-126,^100^ and salivary exosomal miR-10b-5p^105,106^ expression levels were reduced, as they are tumor suppressors. Through the in vitro culturing of OSCC cell lines, miR-365 and other miRNAs are produced in exosomes aberrently^107^ and can be used to assess human papilloma virus (HPV) involvement.^101^ Tissue-derived exosomal circRNA_047733 can be used to indicate the lymph node metastasis (LNM) outcomes of OSCC cases with satisfying specificity and sensitivity.^108^ The in vivo application of these biomarkers necessitates further research. CirRNAs are back-splicing formed RNAs that influence protein translation through interacting with miRNAs, RNA-binding proteins, and RNA Pol.^109^ Serum exosomal circ_0000199 showed a higher level in OSCC patients and is associated with the Tumor Node Metastasis (TNM) stage and prognosis.^110^

Regarding the protein cargos of the exosomes, there are notable differences between the OSCC patients and healthy individuals. TGFβ, a well-known cancer biomarker, provides much more diagnostic and prognostic information for OSCCs in an exosomal form than in a soluble form.^111^ The level of the sera and salivary exosomal marker Alix increases significantly in OSCC patients, but the sensitivity in early cancer (stage I) detection is not satisfying. Moreover, exoAlix behaves differently in sera and saliva in a stage-dependent manner; only serum exoAlix presents prognosis information.^112^ In addition to single-exosomal protein biomarker detection, Li et al.^113^ demonstrated the advantages of combined exosomal C-reactive protein (CRP), von Willebrand factor (VWF), and leucine-rich alpha-2-glycoprotein (LRG) in the determining specificity and sensitivity of early OSCCs diagnosis. The serum exosomal platelet factor 4 variant (PF4V1), C-X-C motif chemokine (CXCL7), coagulation factor XIII, A (F13A1) and Apolipoprotein A-I (ApoA1) not only enable discrimination between the OSCC cases and healthy controls, but also provide information on the lymph node metastasis status. The combination of these four biomarkers could also increase the preciseness.^114^

### Biomarkers for other oral diseases

Exosomal biomarkers have also been discovered in other oral diseases. Viral infectious hand, foot, and mouth disease (HFMD) mostly affects young patients under 5 years old with herpes.^115^ In the sera of patients with HFMD (both the mild and severe types), there is an elevated level of exosomal miR-16-5p, while the miR-671-5p and miR-150-3p levels were decreased.^116^ These statistical data demonstrated the satisfying sensitivity and specificity of exosomes as biomarkers in HFMD diagnosis.

Oral lichen planus (OLP) is an immune-related mucosa disease that is well recognized as a potentially malignant disorder.^117^ Therefore, its early diagnosis would inhibit the progression toward oral cancers and benefit the patients’ prognosis. The exosomal alteration in fluids has provided valuable information regarding OLP detection. Based on the evidence from the polymerase chain reaction (PCR) technique, the serum exosomal miR-34a-5p^118^ and salivary exosomal miR-4484^119^ were significantly upregulated in the OLP samples. Furthermore, the human cytomegalovirus (HCMV)-encoded miR-UL59 manifested a higher level in the OLP samples,^120^ suggesting the underlying connections between HCMV infection and the unclear etiology of OLP.

As an autoimmune disease related to the salivary glands, Sjögren’s syndrome causes alterations in various contents of patients’ fluids. Since 2010, scientists have reported the potential of exosomal miRNA biomarkers used for diagnosing Sjögren’s syndrome.^121^ Novel sequencing evidence has revealed that the levels of exosomal circRNAs circ-IQGAP2 and circ-ZC3H6 increased in the serum samples from primary Sjögren’s syndrome patients.^122^ In addition, in murine models, the levels of serum exosomal miRNA-127-3p, miRNA-409-3p, miRNA-410-3p, miRNA-541-5p, and miRNA-540-5p were upregulated.^123^ These findings deserve further research on the underlying mechanism via human studies.

### Analysis technique for exosomes

**Fig. 2:**
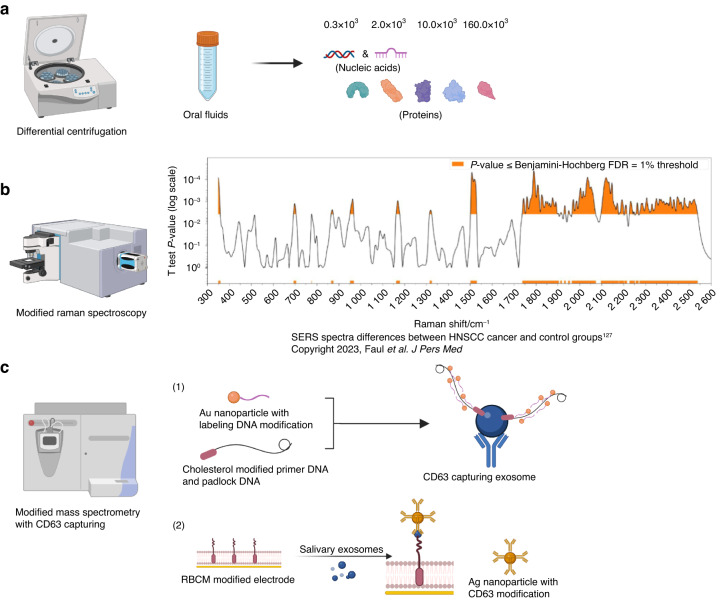
Schematic illustration of exosomal detection techniques. **a** Differential centrifugation can isolate exosome cargos at different fractions. **b** Surface enhancement Raman spectroscopy (SERS) demonstrates distinct Raman spectra between saliva samples from HNSCC and healthy control groups. **c** Modified mass spectrometry with CD63 capturing allows for quantitative analysis of exosomes. **c**-1 Detection of exosomal signal is achieved through cholesterol-based rolling circle amplification and gold-nanoparticle-labeled DNA. **c**-2 Red blood cell membrane (RBCM)-modified electrode can produce electrochemical signals of exosomes with Au nanoparticles

Prior to our evaluation of exosomal biomarkers, the primary focus was the development of efficient and precise detection techniques. The separation and quantitative analysis of exosomes serve as crucial steps in clinical applications.^124^ Centrifugation is the conventional method used for exosome separation. To improve efficiency, differential centrifugation is the most commonly used and practical technique. This approach allows for the isolation of nucleic acid cargos at fractions of 0.3×10^3^ and 2.0×10^3^, while protein cargos appear at different fractions; Alix predominantly appears at the 160.0×10^3^ fraction, and HSP70 exhibits an even distribution across a wide range from 0.3×10^3^ to 160.0×10^3^^124,125^ (Fig. 2a).

The requirements for exosomal quantitative analysis vary depending on specific objectives, such as particle enumeration, protein quantification, RNA quantification, etc.^124^ Consequently, the relevant methodologies include nanoparticle tracking analysis (NTA) and flow cytometry for particle enumeration, bicinchoninic acid (BCA) assaying and PAGE-SDS staining for protein quantification.^124^ Aiming at enhanced efficiency, other researchers have come up with more practical and precise methods for oral exosomal quantitative analysis. Surface enhancement Raman spectroscopy (SERS) is a modified form of Raman spectroscopy (the inelastic scattering of light), which shows an amplified vibrational Raman spectrum when the testing sample is in close proximity to a plasmonic nanostructured surface (Fig. 2b). Based on the analysis of saliva exosomes, SERS exhibits exceptional sensitivity and specificity in distinguishing malignant and normal ones, enabling the early diagnosis of head and neck cancers.^126^ Cheng et al. introduced a new technique for inductively coupled plasma mass spectrometry, performing excellently in oral exosomes quantitative analysis.^127^ They captured exosomes with the CD63 antibody and detected a signal with cholesterol-based rolling circle amplification and gold-nanoparticle-labeled DNA (Fig. 2c-1). Also associated with CD63 capturing, red blood cell membrane (RBCM)-modified electrode could produce electrochemical signals once confronting exosomes, showing great precision in saliva exosomes detection^128^ (Fig. 2c-2).

Although significant progress has been made in the identification of exosomal biomarkers for diagnosing oral diseases, certain limitations still exist. The majority of studies lack sufficient evidence due to inadequate sample collection and classifications for exosomal biomarkers. These deficiencies pose challenges in standardizing their clinical applications. Furthermore, the techniques used for isolating and analyzing exosomes from human fluids are impractical for most basic medical institutions, and a valid consensus has not yet been reached. To address these issues, it is imperative to further develop technical capabilities and gain deeper insights into the role of exosomes in the progression of oral diseases, which will undoubtedly inspire more reliable diagnostic standards.

## Exosomes in targeted therapy for oral diseases

Over recent years, exosomes have been extensively investigated in the treatment of multiple oral diseases. The primary research areas encompass the utilization of engineered exosomes as an innovative drug delivery tool with specific cargos and the clinical application of exosomes derived from orofacial stem cells in tissue regeneration. In this session, we summarize the current research on exosomal therapies toward infections, oral cancers, and the regeneration of pulp and bone.

### Engineered exosomes

#### Anti-infection

Infectious pathogens play a significant role as causative agents and risk factors in various oral diseases. Recent studies have unveiled the potential of the application of exosomes as innovative therapies against microbial infections. An infection with Enterovirus 71 (EV-71) serves as the primary trigger for HFMD, leading to an elevated level of exosomes in the patients’ sera samples.^116^ Consequently, the utilization of the exosome inhibitor GW4869 could effectively reduce infectious activities.^129^ Exosomal miR-155 exhibits a comparable antiviral effect by targeting the phosphatidylinositol clathrin assembly protein (PICALM).^130^ As the first inhabitant microbes in the oral cavity, streptococci prominently influence oral health status, where dysbacteriosis may lead to caries and other oral inflammatory-related diseases. The exosomes separated from honey contain antimicrobial agents that have more potent effects on *Streptococcus mutans* in comparison to those of the other strains.^131^

#### Anti-cancer

Traditional chemo-/immunotherapies for OSCCs are often non-specific to the malignancy and incompatible with the host tissue. However, exosomes, which act as physiological “packages” between cells, may offer a solution to these issues.^132^ The application of engineered exosomes in OSCC treatment can disrupt various processes involved in oncogenesis and tumor microenvironment modulation, thereby effectively inhibiting cancer progression. According to recent studies, many cargos of exosomes attenuate the oncogenesis of OSCCs through complex signaling pathways^133–138^ (Table 2).

**Table 2:** Exosomal cargos exhibiting anti-tumor effect on OSCCs

Exo-cargo	Origin	Function	Mechanism	Research model	Ref
miR-1294	OSCC tissue	Inhibiting OSCC cells proliferation and migration	Targeting oncogene c-Myc	OSCC cell lines	^134^
miR-101-3p	Bone marrow mesenchymal stem cells (BMSCs)	Inhibiting OSCC occurrence and progression	Targeting collagen type X alpha 1 chain (COL10A1)	OSCC cell lines Nude mice	^135^
miR-6887-5p	Eldecalcitol (ED71)-induced OSCC cells	Inhibiting OSCC cells proliferation	Targeting Heparin-binding protein 17/fibroblast growth factor–binding protein-1 (HBp17/FGFBP-1)	OSCC cell lines Nude mice	^136^
miR-34a	Engineered exosomes from HEK-293T cells with miR-34a loading	Inhibiting OSCC cells proliferation, migration, and invasion	Targeting special AT-rich sequence-binding protein 2 (SATB2)	OSCC cells-HN6	^137^
circRNA GDP dissociation inhibitor 2 (circGDI2)	Engineered exosomes from OSCC cells with circGDI2 loading	Inhibiting OSCC cells proliferation, migration, invasion and glycolysis	Regulating miR-424-5p and suppressor of cancer cell invasion (SCAI)	OSCC cell lines BALB/c mice	^138^
LncRNA LBX1-AS1	Immunoglobulin kappa J region (RBPJ) overexpressed macrophages	Inhibiting OSCC cells proliferation, and invasion	Regulating miR-182-5p/FOXO3 pathway	OSCC cell lines Nude mice	^133^

Engineered exosomes secreted from diverse cell types exert inhibitory effects on OSCC development through administrating different drugs or molecules. Macrophage-derived exosomal miR144/451 (tumor suppressive) connected with chitosan nanoparticles exhibit an anti-tumor effect in OSCCs.^139^ The exosomes secreted by menstrual stem cells performed anti-angiogenesis in OSCCs.^140^ In addition to host-derived exosomes, other researchers have also explored alternative sources of therapeutic exosomes for OSCC treatment. Milk exosomes are widely recognized for their exceptional resilience in acidic conditions within the digestive system and their ability to traverse physiological barriers.^141^ The engineered exosomes of milk combined with doxorubicin and an anthracene endoperoxide derivative demonstrate remarkable efficacy in eradicating OSCCs cells,^142^ showing great potential in clinical applications.

#### Novel delivery system based on engineered exosomes

Besides the dental tissue-derived and natural original exosomes mentioned above, engineered exosomes have demonstrated great value as a novel delivery system. The procedure of exosome engineering mainly consists of cargo encapsulation and surface modification, with each step encompassing various methodologies and indications.^143^ Exosomal cargo packing could be induced in situ by interactions between the cargos and donor cell components or in vitro after exosome purification.^132^ Generally, the in vitro methods show more flexibility and practicality in applications, including electroporation, incubation, sonication, extrusion, etc.^80^ In OSCC treatment, Epstein–Barr Virus Induced‑3 (EBI3) transfected fibroblasts were electroporated with anti-tumor small interfering RNAs (siRNAs), the productive engineered exosomes of which significantly targeting OSCCs cells by diminishing their proliferations.^144^ The incubation of miRNA-34a with HEK-293T cells also acquires effective exosomes against OSCCs.^137^

Surface modification is another important step in exosome engineering, aiming at enhancing exosomal targeting toward certain receptors. Multiple components can be added to the exosomal membrane, such as proteins, antigens, antibodies, and DNA/RNA aptamers.^132^ The relevant research has proved that the surface-modified exosomes with peptides can cross blood–brain barrier and treat cerebral ischemia.^145,146^ Although similar research on oral medicine is underway. However, exosome-mimetic nanoparticles have received great attention in oral cancer treatment for their convenience in surface engineering.^147,148^ These biomimetic particles could resemble membrane structures of multiple host cells (blood and stem cells) and escape from immune clearance.^148^

### Dental stem cell-derived exosomes in regenerative therapy

Dental stem cells (DSCs) refer to a group of primitive cells derived from dental tissue, with the potential to proliferate and differentiate. DSCs are generally classified into dental pulp stem cells (DPSCs), stem cells from human exfoliated deciduous teeth (SHED), stem cells from apical papilla (SCAPs), PDLSCs, dental follicle cells (DFCs), and oral mesenchymal stem cells (OMSCs), based on their distinct histologic origin.^149–151^ The application of DSCs in oral regeneration treatment has long been embraced, and stem cell-derived exosomes have recently shown great promise for healing dental defects and orofacial tissues.

#### Pulp regeneration

Due to infectious or traumatic etiologies, pulpal and/or apical diseases invariably result in the irreversible impairment of blood, neural, and nutrient supplies to the natural teeth.^152^ Despite the well-established efficacy of root canal treatment (RCT), the quest for achieving biological pulp regeneration with complete physiological functionality remains ceaseless. There are a series of crucial steps in achieving dental pulp regeneration and forming a physiological pulpodentinal complex, for instance, differentiation from MSCs to functional dental pulp cells (DPCs), the promotion of angiogenesis, and the facilitation of neural reconstruction.^153^ Such as stem cells from pulp tissue, DPSC-derived exosomes positively stimulated the differentiation of stem cells toward DPCs through the P38/MAPK^154^ and miR‑150‑Tlr4 pathways.^155^ Meanwhile, Schwann cells are recruited to enhance neurogenesis with the presence of DPSC-derived exosomes, particularly under the condition of lipopolysaccharide (LPS) stimulation.^156^ LPS also promotes angiogenesis via DPSC-derived exosomes,^157–159^ which simultaneously could be enhanced by hypoxia with higher level of lysyl oxidase-like 2 (LOXL2).^160,161^ Compared with a normal status, DPSCs under odontogenic differentiation condition secrete more effective exosomes, for instance, the levels of miR-27a-5p are elevated, which induces DPSC differentiation.^154,162^ Furthermore, the evidence suggested that younger donors of DPSCs gave better performance to exosomal ability in pulp regeneration,^163^ as exosomal miR-26a secreted by aggregating stem cells from deciduous teeth (SHED) strongly promotes the angiogenesis of HUVECs in pulp tissue through TGF-β/Smad2/3 signaling.^164^ Moreover, the exosomes derived from dental pulp tissue (DPT) exhibit superior efficacy in modulating SCAPs for pulp regeneration compared to that of the DPSCs, which is attributed the “cell-homing technique“.^165^ SCAPs can also release exosomes, facilitating an anti-inflammatory effect on pulpitis during Treg conversion and the dentinogenic differentiation of MSCs,^166,167^ which demonstrate great potential as pulp regenerative therapies.

Besides the DSCs, there are several other stem cells that secrete functional exosomes in pulp regeneration. Embryonic stem cell (ESC)-derived exosomes promote DPCs maturation through CD73 (a type of nucleotidase)-mediated AKT/ERK pathway activation.^168^ Furthermore, the exosomes from umbilical cord mesenchymal stem cells (UCMSCs) showed a great effect on inflammatory alleviation after a pulp injury.^169^ And platelet-sourced exosomes also have the potential for pulp regeneration with thrombin activation.^170^

#### Orofacial bone regeneration

The conventional therapeutic sequence for periodontitis encompasses plaque control, re-evaluation, and surgical intervention. Regenerative surgery has emerged as a pioneering approach in clinical practice, aiming to restore periodontal tissue and regain functions.^171^ Alongside various biofilm and bone graft materials, recent studies have highlighted the potential of exosomal agents for orofacial bone reconstruction.^172^ Controlling the anomaly inflammatory changes in periodontal cells and the microenvironment is the premise of periodontitis regenerative treatment.^11^ Exhibiting periodontal anti-inflammatory effect, gingival mesenchymal stem cell (GMSC)-secreted exosomes target NF-κB signaling and Wnt5a in a periodontal microenvironment^173,174^ and modulate macrophage polarization in high-lipid-level^175^ or TNF-α pre-condition circumstances.^176^ A similar macrophage transformation also occurs with chitosan hydrogel-engineered DPSC-derived exosomes via miR-1246.^177^ In addition, MSC-derived exosomal miR-1246^178^ and PDLSC-derived exosomal miR-155-5p^179^/miR-205-5p^180^ inhibit inflammation by balancing the Th17/Treg ratio.

Alveolar bone loss is a typical manifestation of periodontitis. How to promote bone repair and regeneration is a key issue in periodontitis management. PDLSCs are a group of stem cells residing in the periodontium. The available evidence strongly supports that PDLSCs possess a robust self-renewal capacity and multipotential differentiation abilities. In periodontal regeneration, PDLSCs can differentiate into fibroblasts, osteoblasts, cementoblasts, etc.^181^ During these processes, various stem cells become the sources for exosomes, facilitating PDLSC proliferation and differentiation. Bone marrow mesenchymal stromal cell (BMSC)-derived exosomes have been applied with hydrogel in vivo,^182^ and SHED-derived exosomes were tested in vitro,^183^ both of which showed a promising effect on PDLSCs proliferation, migration, and differentiation. DFC-derived exosomes exhibited a higher efficiency with LPS stimulation through an ROS-mediated antioxidant mechanism in PDLSCs.^184,185^ UCMSC- and PDLSC-derived exosomes are able to activate PDLSCs’ functions even in high-glucose-level circumstances.^186–188^ The application of MSCs enhances periodontal ligament (PDL) cell activation after impairment via exosomes through the AKT/ERK pathway.^189^ In addition, adipose-derived stem/stromal cells (ADSCs) and DFCs exosomes performed a periodontal healing function in murine periodontitis models with newly formed PDL and alveolar bone.^190,191^ Nevertheless, there is a paucity of data related to the underlying mechanisms and applications in human models.

In addition to enhancing the differentiation of PDLSCs, there are also other methods used to promote orofacial bone regeneration, such as the direct activation of osteoblasts and the induction of BMSC osteogenesis. PDLSC-derived exosomes are capable of inducing osteoblast activation,^192^ while osteogenic-induced and differentiated PDLSCs can accelerate BMSC differentiation toward osteoblasts via significant exosomal miRNAs alteration, targeting various osteogenic-related signaling pathways, such as the MAPK and AMPK pathways.^193^ In addition, DPSC-derived exosomes induced jaw bone regeneration in vivo,^194^ and SHED-derived exosomes are capable of promoting naïve BMSCs’ differentiation into osteoblasts.^195–197^ While SHED-derived exosomes can promote DPSCs’ osteogenesis by regulating the mitochondrial transcription factor A (TFAM).^198^ To strengthen the bone repair effect, BMSCs can be innovatively engineered with overexpressed bone morphogenetic protein 2 (BMP2) or miR-26a cargo. These functional modifications significantly improve bone regeneration through the exosomes, targeting the BMP2-associated signaling cascade/mTOR pathway.^199,200^ Moreover, immune modifications performed by engineered exosomes also promote bone regeneration. Dendritic cell-derived exosomes loaded with IL-10 and TGF-β can inhibit immune-related bone resorption and suppress bone loss.^201^ The exosomes secreted by M2 macrophages can be engineered with melatonin, exhibiting anti-inflammatory behavior and rescuing PDLSC potency in differentiation.^202^

Besides periodontitis, there are also other oral diseases characterized by bone destruction that necessitate exosomal regenerative therapies. Osteoarthritis (OA) is a common bone degenerative disease that mainly occurs in joints with cartilage degradation.^203^ The temporomandibular joint (TMJ), as the key joint enabling mandible movement, significantly influences chewing and pronouncing functions. Therefore, temporomandibular joint osteoarthritis (TMJOA) causes severe pain and inconvenience among patients.^204^ Similar to the mechanism of orofacial bone regeneration, the aims of exosomal therapies for TMJOA are reducing the inflammatory response and inducing chondrocyte differentiation.^205^ The SHED-derived exosomal miR-100-5p downregulates the inflammatory factors (IL-6, IL-8, and MMP1) in TMJOA by targeting mTOR signaling.^206^ Moreover, MSC-derived exosomes enhanced the overall cartilage regeneration, and this mechanism is associated with the adenosine activation of the AKT, ERK, and AMPK signaling pathways.^205^

#### Salivary gland revitalization

Exosomal regenerative therapies have also been developed to treat other oral diseases. The dysfunction of the salivary gland may occur in Sjogren’s syndrome, menopause, diabetes, or after radiotherapy for OSCC patients. In vivo studies of exosomal therapies for salivary gland recovery have been conducted using murine models. The DPSC-derived exosomes rescued salivary gland epithelial cells through the G-protein coupled estrogen receptor (GPER)-mediated cAMP/PKA/CREB pathway in Sjogren’s syndrome.^207^ And tonsil mesenchymal stem cell (T-MSC) exosomes contribute to the regaining of salivary gland function after an ovariectomy, resembling the menopause period.^208^ The application of BMSC-derived exosomes could reduce the salivary gland complications in diabetes via the TGF-β/Smad3 pathway,^209^ while the exosomes from the salivary gland performed a similar effect via an unclear mechanism.^210^ Urine-derived stem cells (USCs) under hypoxia stimuli secrete exosomes to repair the salivary gland after radiotherapy via the Wnt3a/GSK3β pathway.^211^

#### Skin regeneration based on anti-aging effect

Skin senescence is a progressive process, with the declining proliferation of cells, reducing ECM, and decreasing repair ability resulting in skin dysfunction. Both intrinsic and extrinsic factors (smoking/ultraviolet light) could influence the skin senescence procedure.^212^ This issue has garnered significant public attention, particularly in the orofacial area, due to its profound impact on patients’ esthetic appearance and functional needs. Novel research has proved the potential applications of stem cell-derived exosomes in skin regeneration based on their anti-aging effects.

The dermis, the layer of skin under the epidermis, mainly consists of the ECM, which is regulated by dermal fibroblasts. As a long-lived cell type, dermal fibroblasts can be used to indicate the skin senescent status through accumulated damage and repair.^213^ Induced pluripotent stem cell (iPSC)-derived exosomes exhibit significant anti-aging effects on dermal fibroblasts, which manifests as the downregulated level of senescence-associated-β-galactosidase (SA-β-Gal) and MMP1/3 and the restoration of collagen type I.^214^ In aged murine models with wounds, the exosomes isolated from young donor wound edge fibroblasts can facilitate fibroblasts differentiation through miR-125b, inhibiting sirtuin 7 (Sirt7).^215^ Similarly, trophoblast-derived exosomes can activate dermal fibroblasts as well.^216^

Wound healing is an intricate process including an inflammatory response, stem cell differentiation and proliferation, ECM modulation, etc.^217^ Senescent skin cells can perform SASP, inducing vicinal inflammation and postponing the healing process.^218^ A pressure ulcer, defined as the localized damage of skin tissue due to a combination of shear and friction,^219^ commonly appears in the orofacial area. A recent survey has suggested the high incidence of facial pressure ulcers in patients with respiratory destruction (such as COVID-19) due to staying prone.^220^ ESC-derived exosomes can rejuvenate epithelial cells and promote the angiogenesis process in aged murine models of pressure ulcers. This mechanism is associated with enriched miR-200a cargo and activated nuclear factor-like 2 (Nrf2) signaling.^221^

Besides the aging factor, the SASP also plays a role in several endocrine diseases, including diabetes.^222^ Diabetic wounds commonly appear on oral soft and hard tissues, which struggle to heal, leading to great suffering.^223^ Therefore, diabetic wound healing requires anti-aging therapies as well, and stem cell-derived exosomes can reduce the SASP. ADSC-derived exosomes accelerated diabetic wound healing, and Nrf2 overexpression enhanced this effect.^224^ A novel biomaterial, oxygen-releasing, antioxidant wound dressing, OxOBand, loaded with ADSC-derived exosomes has been applied in murine diabetic models and shown great performances.^225^ Fetal mesenchymal stem cells (fMSC) can also promote diabetic wound healing through exosomes.^226^ Of note, dental stem cells, such as SHED-derived exosomes, have been used for tendon regeneration, with a significant anti-aging effect through NF-κB inhibition,^227^ which has encouraged us to expand the application of oral original exosomes into other fields.

#### Scaffolds for exosomes in oral regenerative therapy

In addition to exploring the novel sources of exosomes for oral tissue regeneration, it is crucial to consider appropriate scaffolds that interact synergistically with the exosomes in order to optimize the therapeutic efficacy. According to previous studies on pulp regenerative treatment with stem cells, an injectable hydrogel is convenient as a biomaterial applied to the root canals. Different types of scaffolds (such as natural collagen-based scaffolds and synthetic/hybrid materials) with injectable hydrogel have been investigated.^228^ In terms of the scaffolds loaded with exosomes, we should widen our scope to meet the new demands for exosomal applications. Generally, DPSC-derived exosomes can bind to collagen type I and fibronectin, thereby connecting with the biomaterials and promoting DPSC differentiation.^154^ However, these findings were not completed with a certain implementable biomaterial system. Furthermore, the hydrogels engineered with hydroxypropyl chitin (HPCH)/chitin whisker (CW) and the hydrogels with fibrin can both facilitate attraction between the exosomes and MSCs, showing injectable and biocompatible behaviors, ultimately accelerating the exosomes’ effect on pulp regeneration.^229,230^ The controlled releasement of exosomes is also a promising prospect for bio-scaffolding. Poly(lactic-co-glycolic acid) (PLGA)-based biodegradable microspheres have been recently developed to have continuous exosomal effects on pulp regeneration.^231^

In orofacial bone regeneration, the traditional scaffolds mostly focus on mimicking the extracellular matrix (ECM) of natural bones, aiming at enhancing MSC adhesion and osteogenic differentiation.^232^ With a further understanding of the role of exosomes in bone regeneration, new demands for bio-scaffolds are emerging in cell-free therapies. Lyophilized BMSC-derived exosomes on hierarchical mesoporous bioactive glass (MBG) can satisfy both bioactive maintenance and the continuous releasement of exosomes.^233^ In vitro experiments also proved that titanium nanotubes loaded with BMP2-stimulated macrophage-derived exosomes upregulate osteogenic marker (such as alkaline phosphatase) expression.^234^ As a biodegradable polymer that has been widely accepted in controlled delivery,^235^ PLGA has a great performance in exosomal bone regeneration.^236^ Poly-dopamine (pDA)-modified PLGA can ensure the controlled release of exosomes from adipose-derived stem cells (ADSCs), significantly enhancing skull bone repair in murine models.^237^ Combined with metal-organic framework (MOF), the PLGA/Exo-Mg^2+^-gallic acid (GA) system provides the advantages of ADSC-derived exosomes, Mg^2+^ and GA in the anti-inflammation, angiogenesis, and osteogenic differentiation of bone regeneration.^238^ Moreover, adding VEGF and DPSC-derived exosomes to an injectable chitosan nanofibrous microsphere-based PLGA-poly(ethylene glycol) (PEG)-PLGA hydrogel strongly promotes angiogenesis and osteogenesis.^239^ In recent decades, the production of three-dimensional (3D) scaffolds has been widely used in exosomal regenerative medicine.^240^ Three-dimensional-printed silk fibroin/collagen I/nano-hydroxyapatite (SF/COL-I/nHA) scaffolds loaded with UCMSC-derived exosomes could stimulate alveolar bone defect repair in murine models.^241^ In addition, novel scaffolds can also enhance cartilage regeneration. Lithium-substituted bioglass ceramic (Li-BGC) significantly promoted the BMSC-derived exosomal effect on chondrogenesis.^242^

Based on the convincing evidence that DSC-derived exosomes contribute to various oral regenerative therapies, more research is underway. The expansion of MSC-derived exosomes from other tissue origins may benefit oral therapies. And the crosstalk between oral and other diseases, such as general OA^243^ and TMJOA, should be highlighted. Overall, the application of exosomes is one of the key points in oral regenerative treatment. Basic-to-clinic translation is one of the future focuses.

## Conclusions and prospects

**Fig. 3:**
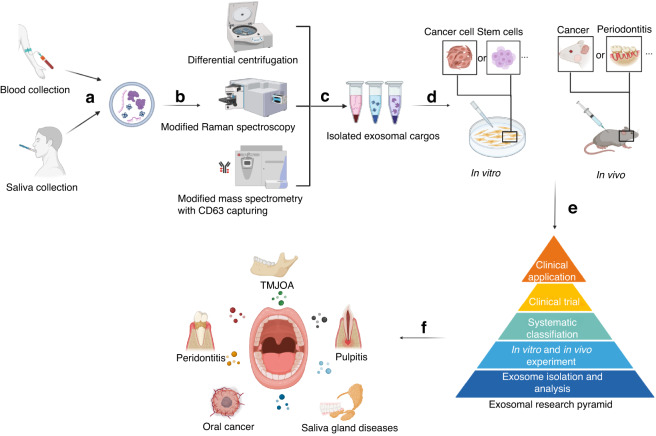
Research flow for exosomes in oral diseases. Exosomes can be obtained from human fluids, such as saliva collected from the oral cavity, providing non-invasive methods for disease detection (**a**). Subsequently, exosomes are isolated from diverse sources, separated and analyzed by novel techniques (e.g., modified centrifugation) (**b**, **c**). The distinct effects of exosomes are then examined through in vitro and/or in vivo experiments (**d**). These comprehensive research findings contribute to the development of a systematic understanding of exosomes, serving as a foundation for clinical applications (**e**). Finally, with a well-established experimental basis, exosomes meet practical clinical applications in oral medicine (**f**)

Exosomes have emerged as a novel research frontier, with a significant application potential across diverse fields. In the realm of oral medicine, exosomes hold promise as non-invasive biomarkers for early disease diagnosis by distinguishing between pathological and healthy states, with periodontitis and OSCCs as the most convincing examples. Furthermore, exploring the distinct characteristics and alterations in exosomes during the progression of oral diseases can deepen our understanding of their underlying pathological mechanisms and pave the way for targeted therapeutic interventions and treatment efficacy. Stem cell-related studies and applications are currently at the forefront of medical research, particularly in relation to regenerative treatments for oral diseases. This intersection with exosome biology provides a valuable foundation for the rational utilization of stem cells (Fig. 3).

Despite the existing research and applications of exosomes in oral medicine, there are still several limitations. First, the identification, analysis, and synthesis of oral exosomes are not precise or convenient enough. In accordance with the updated guidelines for exosomal research^244^ and the specific characterization of the oral cavity (such as the availability of saliva), it is imperative to develop more advanced techniques that will undoubtedly expand the applications of exosomes in oral diseases. Reducing the technique barriers is also important for the promotion of exosomes in real-world medicine, especially in large-scale production and stable storage-to-transportation strategies. Exosomal biomimetic materials might also be promising, combining the inherent advantages of natural exosomes with industrial synthesis techniques. Meeting these diverse demands necessitates collaborative efforts across the biomedical field, while we should raise specific views based on oral diseases properties. Second, there is still lack of clear catalog of the exosomes in oral diseases. Though the current studies involve exosomes in various aspects of oral medicine, they are not yet systematic. While exosomes’ classification primarily relies on their origin, it is worth considering alternative approaches, such as their targeting specific signaling pathways or exploring their distinct effects on cellular functions. With more insights into the abundant exosomal roles throughout oral disease progression and treatment, we ought to find general paths for the future exploration of novel exosomes. These avenues necessitate a deeper understanding of both the exosomes themselves and the underlying mechanisms involved in oral diseases. Third, clinical trials on exosomes in oral diseases are still scarce. According to a relevant analysis, the clinical trials on exosomes have mainly fallen under the respiratory research category, such as biomarkers for lung cancer and therapies for SARSCoV-2 pneumonia.^245^ The primary challenges in conducting clinical trials on exosomes for oral diseases pertain to establishing standardized production criteria and determining the precise dosages for specific indications. Therefore, future studies should prioritize quality control measures and expand the experimental models to more advanced animals. In addition, investigating the crosstalk between oral diseases and other systemic diseases focusing the exosome’s communications could provide valuable insights for future research.

In summary, while the current research on exosomes in oral medicine has yielded significant achievements, there is a substantial amount of work to be conducted. Future studies should not only focus on the physiological and pathological molecular mechanisms associated with exosomes, but also emphasize the feasibility of relevant clinical translation, enhancing the exploration and application of efficient and effective exosomal therapies.

## References

[1] Le H (2017). Oral health disparities and inequities in Asian Americans and Pacific Islanders. Am. J. Public Health.

[2] Peres MA (2019). Oral diseases: a global public health challenge. Lancet.

[3] Cheng L, Hill AF (2022). Therapeutically harnessing extracellular vesicles. Nat. Rev. Drug Discov..

[4] Cocucci E, Meldolesi J (2015). Ectosomes and exosomes: shedding the confusion between extracellular vesicles. Trends Cell Biol..

[5] Kalluri R, LeBleu VS (2020). The biology, function, and biomedical applications of exosomes. Science.

[6] Meldolesi J (2018). Exosomes and ectosomes in intercellular communication. Curr. Biol..

[7] Mathieu M, Martin-Jaular L, Lavieu G, Théry C (2019). Specificities of secretion and uptake of exosomes and other extracellular vesicles for cell-to-cell communication. Nat. Cell Biol..

[8] Hessvik NP, Llorente A (2018). Current knowledge on exosome biogenesis and release. Cell. Mol. Life Sci..

[9] van Niel G, D’Angelo G, Raposo G (2018). Shedding light on the cell biology of extracellular vesicles. Nat. Rev. Mol. Cell Biol..

[10] Meyle J, Chapple I (2015). Molecular aspects of the pathogenesis of periodontitis. Periodontol 2000.

[11] Kinane DF, Stathopoulou PG, Papapanou PN (2017). Periodontal diseases. Nat. Rev. Dis. Prim..

[12] Cai, R. et al. The role of extracellular vesicles in periodontitis: pathogenesis, diagnosis, and therapy. *Front. Immunol.* **14**, 1151322 (2023). 10.3389/fimmu.2023.1151322 PMC10126335 37114060

[13] Zhang Z (2020). PDLSCs regulate angiogenesis of periodontal ligaments via VEGF transferred by exosomes in periodontitis. Int. J. Med. Sci..

[14] Xia Y (2021). The miR-223-3p regulates pyroptosis through NLRP3-Caspase 1-GSDMD signal axis in periodontitis. Inflammation.

[15] Chen Z (2022). Oxidative stress state inhibits exosome secretion of hPDLCs through a specific mechanism mediated by PRMT1. J. Periodontal. Res..

[16] Choi J-W, Kim S-C, Hong S-H, Lee H-J (2017). Secretable small RNAs via outer membrane vesicles in periodontal pathogens. J. Dent. Res..

[17] Elsayed R (2023). Microbially-induced exosomes from dendritic cells promote paracrine immune senescence: novel mechanism of bone degenerative disease in mice. Aging Dis..

[18] Liu, X. et al. Mechanisms of mechanical force aggravating periodontitis: a review. *Oral Dis.*10.1111/odi.14566 (2023). 10.1111/odi.14566 36989127

[19] Wu, Y. et al. Exosomes from cyclic stretched periodontal ligament cells induced periodontal inflammation through miR-9-5p/SIRT1/NF-κB signaling pathway. *J. Immunol.*10.4049/jimmunol.2300074 (2023). 10.4049/jimmunol.2300074 37154707

[20] Zhao M, Ma Q, Zhao Z, Guan X, Bai Y (2021). Periodontal ligament fibroblast-derived exosomes induced by compressive force promote macrophage M1 polarization via Yes-associated protein. Arch. Oral. Biol..

[21] Piccolo S, Dupont S, Cordenonsi M (2014). The biology of YAP/TAZ: hippo signaling and beyond. Physiol. Rev..

[22] Matthaios D, Tolia M, Mauri D, Kamposioras K, Karamouzis M (2021). YAP/Hippo pathway and cancer immunity: it takes two to tango. Biomedicines.

[23] Atsawasuwan P (2018). Secretory microRNA-29 expression in gingival crevicular fluid during orthodontic tooth movement. PLoS One.

[24] Zheng, X. et al. Biological characteristics of microRNAs secreted by exosomes of periodontal ligament stem cells due to mechanical force. *Eur. J. Orthod.*10.1093/ejo/cjad002 (2023). 10.1093/ejo/cjad002 37262013

[25] Chang M, Chen Q, Wang B, Zhang Z, Han G (2023). Exosomes from tension force-applied periodontal ligament cells promote mesenchymal stem cell recruitment by altering microRNA profiles. Int. J. Stem Cells.

[26] Huang H-M (2022). Mechanical force-promoted osteoclastic differentiation via periodontal ligament stem cell exosomal protein ANXA3. Stem Cell Rep..

[27] Liu X, Muhammed FK, Liu Y (2022). Simvastatin encapsulated in exosomes can enhance its inhibition of relapse after orthodontic tooth movement. Am. J. Orthod. Dentofac. Orthop..

[28] Sarode G (2020). Epidemiologic aspects of oral cancer. Dis. Mon..

[29] Hernández-Morales A (2023). Lip and oral cavity cancer incidence and mortality rates associated with smoking and chewing tobacco use and the human development index in 172 countries worldwide: an ecological study 2019–2020. Healthcare.

[30] Tan Y (2023). Oral squamous cell carcinomas: state of the field and emerging directions. Int. J. Oral. Sci..

[31] Qadir F (2018). Transcriptome reprogramming by cancer exosomes: identification of novel molecular targets in matrix and immune modulation. Mol. Cancer.

[32] Razzo BM (2020). Tumor-derived exosomes promote carcinogenesis of murine oral squamous cell carcinoma. Carcinogenesis.

[33] Li X, Yang T, Shu C (2022). The oral tumor cell exosome miR-10b stimulates cell invasion and relocation via AKT signaling. Contrast Media Mol. Imaging.

[34] Li C (2022). Exosomal long noncoding RNAs MAGI2-AS3 and CCDC144NL-AS1 in oral squamous cell carcinoma development via the PI3K-AKT-mTOR signaling pathway. Pathol. Res. Pract..

[35] Hanahan D (2022). Hallmarks of cancer: new dimensions. Cancer Discov..

[36] Squarize CH (2013). PTEN deficiency contributes to the development and progression of head and neck cancer. Neoplasia.

[37] Yan W (2021). Exosomal miR-130b-3p promotes progression and tubular formation through targeting PTEN in oral squamous cell carcinoma. Front. Cell Dev. Biol..

[38] Hou C-X (2022). Exosomal microRNA-23b-3p promotes tumor angiogenesis and metastasis by targeting PTEN in salivary adenoid cystic carcinoma. Carcinogenesis.

[39] He S (2021). Oral squamous cell carcinoma (OSCC)-derived exosomal MiR-221 targets and regulates phosphoinositide-3-kinase regulatory subunit 1 (PIK3R1) to promote human umbilical vein endothelial cells migration and tube formation. Bioengineered.

[40] Wang H (2020). OSCC exosomes regulate miR-210-3p targeting EFNA3 to promote oral cancer angiogenesis through the PI3K/AKT pathway. BioMed. Res. Int..

[41] Li S (2022). Mesenchymal stem cell-exosome-mediated matrix metalloproteinase 1 participates in oral leukoplakia and carcinogenesis by inducing angiogenesis. J. Oral. Pathol. Med..

[42] Hinshaw DC, Shevde LA (2019). The tumor microenvironment innately modulates cancer progression. Cancer Res..

[43] Mito I (2023). Tumor-derived exosomes elicit cancer-associated fibroblasts shaping inflammatory tumor microenvironment in head and neck squamous cell carcinoma. Oral. Oncol..

[44] Li C, Teixeira AF, Zhu H-J, Ten Dijke P (2021). Cancer associated-fibroblast-derived exosomes in cancer progression. Mol. Cancer.

[45] Sun L-P (2019). Cancer‑associated fibroblast‑derived exosomal miR‑382‑5p promotes the migration and invasion of oral squamous cell carcinoma. Oncol. Rep..

[46] He L (2023). Exosomal miR-146b-5p derived from cancer-associated fibroblasts promotes progression of oral squamous cell carcinoma by downregulating HIPK3. Cell Signal.

[47] Li Y-Y (2018). Cancer-associated fibroblasts contribute to oral cancer cells proliferation and metastasis via exosome-mediated paracrine miR-34a-5p. EBioMedicine.

[48] Kallinger, I. et al. Tumor gene signatures that correlate with release of extracellular vesicles shape the immune landscape in head and neck squamous cell carcinoma. *Clin. Exp. Immunol.*10.1093/cei/uxad019 (2023). 10.1093/cei/uxad019 PMC10324554 36752300

[49] Yunna C, Mengru H, Lei W, Weidong C (2020). Macrophage M1/M2 polarization. Eur. J. Pharm..

[50] Pan Y, Yu Y, Wang X, Zhang T (2020). Tumor-associated macrophages in tumor immunity. Front. Immunol..

[51] Oshi M (2020). M1 Macrophage and M1/M2 ratio defined by transcriptomic signatures resemble only part of their conventional clinical characteristics in breast cancer. Sci. Rep..

[52] Ludwig N (2022). TGFβ+ small extracellular vesicles from head and neck squamous cell carcinoma cells reprogram macrophages towards a pro-angiogenic phenotype. J. Extracell. Vesicles.

[53] Xiao M, Zhang J, Chen W, Chen W (2018). M1-like tumor-associated macrophages activated by exosome-transferred THBS1 promote malignant migration in oral squamous cell carcinoma. J. Exp. Clin. Cancer Res..

[54] You Y (2022). M1-like tumor-associated macrophages cascade a mesenchymal/stem-like phenotype of oral squamous cell carcinoma via the IL6/Stat3/THBS1 feedback loop. J. Exp. Clin. Cancer Res..

[55] Wu L, Ye S, Yao Y, Zhang C, Liu W (2022). Oral cancer stem cell-derived small extracellular vesicles promote M2 macrophage polarization and suppress CD4+ T-cell activity by transferring UCA1 and targeting LAMC2. Stem Cells Int..

[56] Manikandan M (2016). Oral squamous cell carcinoma: microRNA expression profiling and integrative analyses for elucidation of tumourigenesis mechanism. Mol. Cancer.

[57] Cai J, Qiao B, Gao N, Lin N, He W (2019). Oral squamous cell carcinoma-derived exosomes promote M2 subtype macrophage polarization mediated by exosome-enclosed miR-29a-3p. Am. J. Physiol. Cell Physiol..

[58] Pang X (2021). OSCC cell-secreted exosomal CMTM6 induced M2-like macrophages polarization via ERK1/2 signaling pathway. Cancer Immunol. Immunother..

[59] Ono K (2020). Triple knockdown of CDC37, HSP90-alpha and HSP90-beta diminishes extracellular vesicles-driven malignancy events and macrophage M2 polarization in oral cancer. J. Extracell. Vesicles.

[60] Yuan Y (2022). Endoplasmic reticulum stress promotes the release of exosomal PD-L1 from head and neck cancer cells and facilitates M2 macrophage polarization. Cell Commun. Signal.

[61] Kumar P, Bhattacharya P, Prabhakar BS (2018). A comprehensive review on the role of co-signaling receptors and Treg homeostasis in autoimmunity and tumor immunity. J. Autoimmun..

[62] Chen Y (2022). CircRNA has_circ_0069313 induced OSCC immunity escape by miR-325-3p-Foxp3 axes in both OSCC cells and Treg cells. Aging (Albany NY).

[63] Johnson DE (2020). Head and neck squamous cell carcinoma. Nat. Rev. Dis. Prim..

[64] Ramos A, Sadeghi S, Tabatabaeian H (2021). Battling chemoresistance in cancer: root causes and strategies to uproot them. Int. J. Mol. Sci..

[65] Law Z-J (2021). Extracellular vesicle-mediated chemoresistance in oral squamous cell carcinoma. Front. Mol. Biosci..

[66] Cheng H-Y (2022). Snail-regulated exosomal microRNA-21 suppresses NLRP3 inflammasome activity to enhance cisplatin resistance. J. Immunother. Cancer.

[67] Qin X (2019). Exosomal miR-196a derived from cancer-associated fibroblasts confers cisplatin resistance in head and neck cancer through targeting CDKN1B and ING5. Genome Biol..

[68] Tomita R, Sasabe E, Tomomura A, Yamamoto T (2020). Macrophage‑derived exosomes attenuate the susceptibility of oral squamous cell carcinoma cells to chemotherapeutic drugs through the AKT/GSK‑3β pathway. Oncol. Rep..

[69] Sayyed AA (2021). MiR-155 inhibitor-laden exosomes reverse resistance to cisplatin in a 3D tumor spheroid and xenograft model of oral cancer. Mol. Pharm..

[70] Kulkarni B (2020). Exosome-mediated delivery of miR-30a sensitize cisplatin-resistant variant of oral squamous carcinoma cells via modulating Beclin1 and Bcl2. Oncotarget.

[71] Li Y (2022). Irradiated cell-derived exosomes transmit essential molecules inducing radiation therapy resistance. Int. J. Radiat. Oncol. Biol. Phys..

[72] Mutschelknaus L (2016). Exosomes derived from squamous head and neck cancer promote cell survival after ionizing radiation. PLoS One.

[73] Theodoraki M-N (2019). Circulating exosomes measure responses to therapy in head and neck cancer patients treated with cetuximab, ipilimumab, and IMRT. Oncoimmunology.

[74] Theodoraki M-N (2021). Changes in circulating exosome molecular profiles following surgery/(chemo)radiotherapy: early detection of response in head and neck cancer patients. Br. J. Cancer.

[75] Vasey C, McBride J, Penta K (2021). Circadian rhythm dysregulation and restoration: the role of melatonin. Nutrients.

[76] Wang L, Wang C, Choi WS (2022). Use of melatonin in cancer treatment: where are we?. Int. J. Mol. Sci..

[77] Capote-Moreno A (2019). Potential of melatonin as adjuvant therapy of oral cancer in the era of epigenomics. Cancers (Basel).

[78] Hunsaker M, Barba G, Kingsley K, Howard KM (2019). Differential microRNA expression of miR-21 and miR-155 within oral cancer extracellular vesicles in response to melatonin. Dent. J. (Basel).

[79] Nikanjam M, Kato S, Kurzrock R (2022). Liquid biopsy: current technology and clinical applications. J. Hematol. Oncol..

[80] Zeng H (2023). Current strategies for exosome cargo loading and targeting delivery. Cells.

[81] Peng Q, Yang J-Y, Zhou G (2020). Emerging functions and clinical applications of exosomes in human oral diseases. Cell Biosci..

[82] Slots J (2017). Periodontitis: facts, fallacies and the future. Periodontol 2000.

[83] Chaparro Padilla, A. et al. Molecular signatures of extracellular vesicles in oral fluids of periodontitis patients. *Oral Dis.*10.1111/odi.13338 (2020). 10.1111/odi.13338 32232928

[84] Panni S, Lovering RC, Porras P, Orchard S (2020). Non-coding RNA regulatory networks. Biochim. Biophys. Acta Gene. Regul. Mech..

[85] Budakoti M (2021). Micro-RNA: the darkhorse of cancer. Cell Signal.

[86] Nik Mohamed Kamal NNS, Awang RAR, Mohamad S, Shahidan WNS (2020). Plasma- and saliva exosome profile reveals a distinct microRNA signature in chronic periodontitis. Front. Physiol..

[87] Kwon EJ (2023). Profiling of plasma-derived exosomal RNA expression in patients with periodontitis: a pilot study. Oral. Dis..

[88] Han P (2023). TNF-α and OSX mRNA of salivary small extracellular vesicles in periodontitis: a pilot study. Tissue Eng. Part C. Methods.

[89] Micó-Martínez P (2018). miR-1226 detection in GCF as potential biomarker of chronic periodontitis: a pilot study. Med. Oral. Patol. Oral. Cir. Bucal..

[90] Han P, Bartold PM, Salomon C, Ivanovski S (2020). Salivary small extracellular vesicles associated miRNAs in periodontal status—a pilot study. Int. J. Mol. Sci..

[91] Fujimori K (2019). Detection of salivary miRNAs reflecting chronic periodontitis: a pilot study. Molecules.

[92] Yu J (2019). Detection of exosomal PD-L1 RNA in saliva of patients with periodontitis. Front. Genet.

[93] Tobón-Arroyave SI, Celis-Mejía N, Córdoba-Hidalgo MP, Isaza-Guzmán DM (2019). Decreased salivary concentration of CD9 and CD81 exosome-related tetraspanins may be associated with the periodontal clinical status. J. Clin. Periodontol..

[94] Huang X, Hu X, Zhao M, Zhang Q (2020). Analysis of salivary exosomal proteins in young adults with severe periodontitis. Oral. Dis..

[95] Macey, R. et al. Diagnostic tests for oral cancer and potentially malignant disorders in patients presenting with clinically evident lesions. *Cochrane Database Syst. Rev.*10.1002/14651858.CD010276.pub2 (2015). 10.1002/14651858.CD010276.pub2 PMC7087440 26021841

[96] Warnakulasuriya S, Kerr AR (2021). Oral cancer screening: past, present, and future. J. Dent. Res..

[97] Zlotogorski-Hurvitz A, Dayan D, Chaushu G, Salo T, Vered M (2016). Morphological and molecular features of oral fluid-derived exosomes: oral cancer patients versus healthy individuals. J. Cancer Res. Clin. Oncol..

[98] Rabinowits, G. et al. Comparative analysis of microRNA expression among benign and malignant tongue tissue and plasma of patients with tongue cancer. *Front. Oncol.* **7**, 191 (2017). 10.3389/fonc.2017.00191 PMC5581802 28900608

[99] He T (2021). Plasma-derived exosomal microRNA-130a serves as a noninvasive biomarker for diagnosis and prognosis of oral squamous cell carcinoma. J. Oncol..

[100] Chen C-M (2021). Exosome-derived microRNAs in oral squamous cell carcinomas impact disease prognosis. Oral. Oncol..

[101] Galiveti CR (2023). Small extravesicular microRNA in head and neck squamous cell carcinoma and its potential as a liquid biopsy for early detection. Head Neck.

[102] He L (2020). Salivary exosomal miR-24-3p serves as a potential detective biomarker for oral squamous cell carcinoma screening. Biomed. Pharmacother..

[103] Patel A (2022). Salivary exosomal miRNA-1307-5p predicts disease aggressiveness and poor prognosis in oral squamous cell carcinoma patients. Int. J. Mol. Sci..

[104] Farag A, Sabry D, Hassabou N, Alaa El-din Y (2021). MicroRNA-134/microRNA-200a derived salivary exosomes are novel diagnostic biomarkers of oral squamous cell carcinoma. Egypt. Dent. J..

[105] Faur CI (2022). Salivary exosomal microRNA-486-5p and microRNA-10b-5p in oral and oropharyngeal squamous cell carcinoma. Medicina (Kaunas).

[106] Langevin S (2017). Comprehensive microRNA-sequencing of exosomes derived from head and neck carcinoma cells in vitro reveals common secretion profiles and potential utility as salivary biomarkers. Oncotarget.

[107] Coon J, Kingsley K, Howard K (2020). miR-365 (microRNA): potential biomarker in oral squamous cell carcinoma exosomes and extracellular vesicles. Int. J. Mol. Sci..

[108] Deng Q (2023). Exosomal hsa_circRNA_047733 integrated with clinical features for preoperative prediction of lymph node metastasis risk in oral squamous cell carcinoma. J. Oral. Pathol. Med..

[109] Xia S (2017). Comprehensive characterization of tissue-specific circular RNAs in the human and mouse genomes. Brief. Bioinform.

[110] Luo Y, Liu F, Guo J, Gui R (2020). Upregulation of circ_0000199 in circulating exosomes is associated with survival outcome in OSCC. Sci. Rep..

[111] Ludwig N (2023). TGFβ carrying exosomes in plasma: potential biomarkers of cancer progression in patients with head and neck squamous cell carcinoma. Br. J. Cancer.

[112] Nakamichi E (2021). Detection of serum/salivary exosomal Alix in patients with oral squamous cell carcinoma. Oral. Dis..

[113] Guo H, Jiang W, Huang S, Huang X, Li C (2021). Serum exosome-derived biomarkers for the early detection of oral squamous cell carcinoma. Mol. Cell Biochem..

[114] Li C (2019). Potential markers from serum-purified exosomes for detecting oral squamous cell carcinoma metastasis. Cancer Epidemiol. Biomark. Prev..

[115] Zhu P (2023). Current status of hand-foot-and-mouth disease. J. Biomed. Sci..

[116] Jia H-L (2014). MicroRNA expression profile in exosome discriminates extremely severe infections from mild infections for hand, foot and mouth disease. BMC Infect. Dis..

[117] Hamour AF, Klieb H, Eskander A (2020). Oral lichen planus. CMAJ.

[118] Peng Q, Zhang J, Zhou G (2018). Differentially circulating exosomal microRNAs expression profiling in oral lichen planus. Am. J. Transl. Res..

[119] Byun J-S, Hong S-H, Choi J-K, Jung J-K, Lee H-J (2015). Diagnostic profiling of salivary exosomal microRNAs in oral lichen planus patients. Oral. Dis..

[120] Ding M (2017). Distinct expression profile of HCMV encoded miRNAs in plasma from oral lichen planus patients. J. Transl. Med..

[121] Michael A (2010). Exosomes from human saliva as a source of microRNA biomarkers. Oral. Dis..

[122] Li F (2020). Circular RNA sequencing indicates circ-IQGAP2 and circ-ZC3H6 as noninvasive biomarkers of primary Sjögren’s syndrome. Rheumatology (Oxford).

[123] Kakan SS (2020). Small RNA deep sequencing identifies a unique miRNA signature released in serum exosomes in a mouse model of Sjögren’s syndrome. Front. Immunol..

[124] Théry C (2018). Minimal information for studies of extracellular vesicles 2018 (MISEV2018): a position statement of the International Society for Extracellular Vesicles and update of the MISEV2014 guidelines. J. Extracell. Vesicles.

[125] Hiraga C (2021). Pentapartite fractionation of particles in oral fluids by differential centrifugation. Sci. Rep..

[126] Faur CI (2023). A new detection method of oral and oropharyngeal squamous cell carcinoma based on multivariate analysis of surface enhanced raman spectra of salivary exosomes. J. Pers. Med..

[127] Cheng Y (2022). Sensitive detection of exosomes by gold nanoparticles labeling inductively coupled plasma mass spectrometry based on cholesterol recognition and rolling circle amplification. Anal. Chim. Acta.

[128] He L, Shao M, Xu J, Chen H (2021). Engineered red blood cell membrane for sensitive and precise electrochemical detection of salivary exosomes. Anal. Methods.

[129] Ruan Z (2022). Enterovirus 71 non-structural protein 3A hijacks vacuolar protein sorting 25 to boost exosome biogenesis to facilitate viral replication. Front. Microbiol..

[130] Wu J (2019). Exosomal microRNA-155 inhibits enterovirus A71 infection by targeting PICALM. Int. J. Biol. Sci..

[131] Leiva-Sabadini C, Alvarez S, Barrera NP, Schuh CMAP, Aguayo S (2021). Antibacterial effect of honey-derived exosomes containing antimicrobial peptides against oral Streptococci. Int. J. Nanomed..

[132] Liang Y, Duan L, Lu J, Xia J (2021). Engineering exosomes for targeted drug delivery. Theranostics.

[133] Ai Y (2021). Exosomal LncRNA LBX1-AS1 derived from RBPJ overexpressed-macrophages inhibits oral squamous cell carcinoma progress via miR-182-5p/FOXO3. Front Oncol..

[134] Wang Z, Yan J, Zou T, Gao H (2018). MicroRNA-1294 inhibited oral squamous cell carcinoma growth by targeting c-Myc. Oncol. Lett..

[135] Xie C, Du L-Y, Guo F, Li X, Cheng B (2019). Exosomes derived from microRNA-101-3p-overexpressing human bone marrow mesenchymal stem cells suppress oral cancer cell proliferation, invasion, and migration. Mol. Cell Biochem..

[136] Higaki M, Shintani T, Hamada A, Rosli SNZ, Okamoto T (2020). Eldecalcitol (ED-71)-induced exosomal miR-6887-5p suppresses squamous cell carcinoma cell growth by targeting heparin-binding protein 17/fibroblast growth factor–binding protein-1 (HBp17/FGFBP-1). Vitr. Cell. Dev. Biol. Anim..

[137] Deng W (2022). In vitro experimental study on the formation of microRNA-34a loaded exosomes and their inhibitory effect in oral squamous cell carcinoma. Cell Cycle.

[138] Zhang Y, Tang K, Chen L, Du M, Qu Z (2020). Exosomal CircGDI2 suppresses oral squamous cell carcinoma progression through the regulation of MiR-424-5p/SCAI Axis. Cancer Manag Res.

[139] Li K, Qiu Y, Liu X, Huang F (2022). Biomimetic nanosystems for the synergistic delivery of miR-144/451a for oral squamous cell carcinoma. Balk. Med. J..

[140] Rosenberger L (2019). Stem cell exosomes inhibit angiogenesis and tumor growth of oral squamous cell carcinoma. Sci. Rep..

[141] Zhong J (2021). High-quality milk exosomes as oral drug delivery system. Biomaterials.

[142] Zhang Q (2020). Milk-exosome based pH/light sensitive drug system to enhance anticancer activity against oral squamous cell carcinoma. RSC Adv..

[143] Antimisiaris SG, Mourtas S, Marazioti A (2018). Exosomes and exosome-inspired vesicles for targeted drug delivery. Pharmaceutics.

[144] Kase Y (2021). Engineered exosomes delivering specific tumor-suppressive RNAi attenuate oral cancer progression. Sci. Rep..

[145] Tian T (2018). Surface functionalized exosomes as targeted drug delivery vehicles for cerebral ischemia therapy. Biomaterials.

[146] Tian T (2021). Targeted delivery of neural progenitor cell-derived extracellular vesicles for anti-inflammation after cerebral ischemia. Theranostics.

[147] Bai Y-T, Zhang X-Q, Chen X-J, Zhou G (2022). Nanomedicines in oral cancer: inspiration comes from extracellular vesicles and biomimetic nanoparticles. Nanomedicine (Lond.).

[148] Zha Y (2020). Exosome-mimetics as an engineered gene-activated matrix induces in-situ vascularized osteogenesis. Biomaterials.

[149] Zhai Q, Dong Z, Wang W, Li B, Jin Y (2019). Dental stem cell and dental tissue regeneration. Front. Med..

[150] Luo H (2024). Advances in oral mesenchymal stem cell-derived extracellular vesicles in health and disease. Genes Dis..

[151] Morsczeck C (2023). Dental stem cells for tooth regeneration: how far have we come and where next?. Expert Opin. Biol. Ther..

[152] Ordinola-Zapata R, Noblett WC, Perez-Ron A, Ye Z, Vera J (2022). Present status and future directions of intracanal medicaments. Int. Endod. J..

[153] Xie Z (2021). Functional dental pulp regeneration: basic research and clinical translation. Int. J. Mol. Sci..

[154] Huang C-C, Narayanan R, Alapati S, Ravindran S (2016). Exosomes as biomimetic tools for stem cell differentiation: applications in dental pulp tissue regeneration. Biomaterials.

[155] Li L, Ge J (2022). Exosome‑derived lncRNA‑Ankrd26 promotes dental pulp restoration by regulating miR‑150‑TLR4 signaling. Mol. Med. Rep..

[156] Li J, Ju Y, Liu S, Fu Y, Zhao S (2021). Exosomes derived from lipopolysaccharide-preconditioned human dental pulp stem cells regulate Schwann cell migration and differentiation. Connect Tissue Res..

[157] Xian X, Gong Q, Li C, Guo B, Jiang H (2018). Exosomes with highly angiogenic potential for possible use in pulp regeneration. J. Endod..

[158] Huang X (2021). Exosomes from LPS-stimulated hDPSCs activated the angiogenic potential of HUVECs in vitro. Stem Cells Int..

[159] Chen W-J (2021). The role of small extracellular vesicles derived from lipopolysaccharide-preconditioned human dental pulp stem cells in dental pulp regeneration. J. Endod..

[160] Li B (2022). Hypoxia alters the proteome profile and enhances the angiogenic potential of dental pulp stem cell-derived exosomes. Biomolecules.

[161] Li B (2023). Hypoxia preconditioned DPSC-derived exosomes regulate angiogenesis via transferring LOXL2. Exp. Cell Res..

[162] Hu X (2019). Lineage-specific exosomes promote the odontogenic differentiation of human dental pulp stem cells (DPSCs) through TGFβ1/smads signaling pathway via transfer of microRNAs. Stem Cell Res. Ther..

[163] Brunello G (2022). Exosomes derived from dental pulp stem cells show different angiogenic and osteogenic properties in relation to the age of the donor. Pharmaceutics.

[164] Wu M (2021). SHED aggregate exosomes shuttled miR-26a promote angiogenesis in pulp regeneration via TGF-β/SMAD2/3 signalling. Cell Prolif..

[165] Chen Y (2022). The application of pulp tissue derived-exosomes in pulp regeneration: a novel cell-homing approach. Int. J. Nanomed..

[166] Zhuang X (2020). Exosomes derived from stem cells from the apical papilla promote dentine-pulp complex regeneration by inducing specific dentinogenesis. Stem Cells Int..

[167] Yu S (2022). Exosomes derived from stem cells from the apical papilla alleviate inflammation in rat pulpitis by upregulating regulatory T cells. Int. Endod. J..

[168] Shi J (2023). Mesenchymal stromal cell exosomes enhance dental pulp cell functions and promote pulp-dentin regeneration. Biomater. Biosyst..

[169] Zeng J (2022). Exosomes from human umbilical cord mesenchymal stem cells and human dental pulp stem cells ameliorate lipopolysaccharide-induced inflammation in human dental pulp stem cells. Arch. Oral. Biol..

[170] Bagio DA, Julianto I, Margono A, Suprastiwi E (2023). Analysis of thrombin-activated platelet-derived exosome (T-aPDE) potential for dental pulp regeneration: in-vitro study. Eur. J. Dent..

[171] Kwon T, Lamster IB, Levin L (2021). Current concepts in the management of periodontitis. Int. Dent. J..

[172] Elangovan S, Gajendrareddy P, Ravindran S, Salem AK (2020). Emerging local delivery strategies to enhance bone regeneration. Biomed. Mater..

[173] Sun J (2022). Exosomes derived from human gingival mesenchymal stem cells attenuate the inflammatory response in periodontal ligament stem cells. Front. Chem..

[174] Hu Y (2023). Human gingival mesenchymal stem cell-derived exosomes cross-regulate the Wnt/β-catenin and NF-κB signalling pathways in the periodontal inflammation microenvironment. J. Clin. Periodontol..

[175] Zhang Y (2021). Effect of gingival mesenchymal stem cell-derived exosomes on inflammatory macrophages in a high-lipid microenvironment. Int. Immunopharmacol..

[176] Nakao Y (2021). Exosomes from TNF-α-treated human gingiva-derived MSCs enhance M2 macrophage polarization and inhibit periodontal bone loss. Acta Biomater..

[177] Shen Z (2020). Chitosan hydrogel incorporated with dental pulp stem cell-derived exosomes alleviates periodontitis in mice via a macrophage-dependent mechanism. Bioact. Mater..

[178] Zhang Y (2021). Exosomes derived from 3D-cultured MSCs improve therapeutic effects in periodontitis and experimental colitis and restore the Th17 cell/Treg balance in inflamed periodontium. Int. J. Oral. Sci..

[179] Zheng Y (2019). Exosomal microRNA-155-5p from PDLSCs regulated Th17/Treg balance by targeting sirtuin-1 in chronic periodontitis. J. Cell. Physiol..

[180] Kang L, Miao Y, Jin Y, Shen S, Lin X (2023). Exosomal miR-205-5p derived from periodontal ligament stem cells attenuates the inflammation of chronic periodontitis via targeting XBP1. Immun. Inflamm. Dis..

[181] Tomokiyo A, Wada N, Maeda H (2019). Periodontal ligament stem cells: regenerative potency in periodontium. Stem Cells Dev..

[182] Liu L (2021). Bone marrow mesenchymal stem cell-derived small extracellular vesicles promote periodontal regeneration. Tissue Eng. Part A.

[183] Wang M, Li J, Ye Y, Chen D, Song J (2023). SHED-derived exosomes improve the repair capacity and osteogenesis potential of hPDLCs. Oral. Dis..

[184] Shi W (2020). Small extracellular vesicles from lipopolysaccharide-preconditioned dental follicle cells promote periodontal regeneration in an inflammatory microenvironment. ACS Biomater. Sci. Eng..

[185] Huang Y (2022). Lipopolysaccharide-preconditioned dental follicle stem cells derived small extracellular vesicles treating periodontitis via reactive oxygen species/mitogen-activated protein kinase signaling-mediated antioxidant effect. Int. J. Nanomedicine.

[186] Yang S (2022). Exosomes derived from human umbilical cord mesenchymal stem cells enhance the osteoblastic differentiation of periodontal ligament stem cells under high glucose conditions through the PI3K/AKT signaling pathway. Biomed. Environ. Sci..

[187] Lei F (2022). Treatment of inflammatory bone loss in periodontitis by stem cell-derived exosomes. Acta Biomater..

[188] Liu M (2023). Exosomal miR-141-3p from PDLSCs alleviates high glucose-induced senescence of PDLSCs by activating the KEAP1-NRF2 signaling pathway. Stem Cells Int..

[189] Chew JRJ (2019). Mesenchymal stem cell exosomes enhance periodontal ligament cell functions and promote periodontal regeneration. Acta Biomater..

[190] Mohammed E, Khalil E, Sabry D (2018). Effect of adipose-derived stem cells and their exo as adjunctive therapy to nonsurgical periodontal treatment: a histologic and histomorphometric study in rats. Biomolecules.

[191] Ma L (2022). Small extracellular vesicles from dental follicle stem cells provide biochemical cues for periodontal tissue regeneration. Stem Cell Res. Ther..

[192] Lan, Q., Xiao, X., Bi, X., Gu, Y. & Ai, Y. Effects of periodontal ligament stem cell-derived exosomes on osteoblastic proliferation, migration, differentiation, apoptosis, and signaling pathways. *Oral Dis.*10.1111/odi.14375 (2022). 10.1111/odi.14375 36076350

[193] Liu T (2020). Human periodontal ligament stem cell-derived exosomes promote bone regeneration by altering microRNA profiles. Stem Cells Int..

[194] Lee AE (2023). DPSC-derived extracellular vesicles promote rat jawbone regeneration. J. Dent. Res..

[195] Wei J (2020). Exosomes derived from human exfoliated deciduous teeth ameliorate adult bone loss in mice through promoting osteogenesis. J. Mol. Histol..

[196] Luo L, Avery SJ, Waddington RJ (2021). Exploring a chemotactic role for EVs from progenitor cell populations of human exfoliated deciduous teeth for promoting migration of naïve BMSCs in bone repair process. Stem Cells Int..

[197] Wu J (2019). Exosomes secreted by stem cells from human exfoliated deciduous teeth promote alveolar bone defect repair through the regulation of angiogenesis and osteogenesis. ACS Biomater. Sci. Eng..

[198] Guo J (2022). Exosome-shuttled mitochondrial transcription factor A mRNA promotes the osteogenesis of dental pulp stem cells through mitochondrial oxidative phosphorylation activation. Cell Prolif..

[199] Huang C-C (2020). Functionally engineered extracellular vesicles improve bone regeneration. Acta Biomater..

[200] Lai S (2023). Bone marrow mesenchymal stem cell-derived exosomes loaded with miR-26a through the novel immunomodulatory peptide DP7-C can promote osteogenesis. Biotechnol. Lett..

[201] Elashiry M (2020). Dendritic cell derived exosomes loaded with immunoregulatory cargo reprogram local immune responses and inhibit degenerative bone disease in vivo. J. Extracell. Vesicles.

[202] Cui, Y. et al. Melatonin engineering M2 macrophage-derived exosomes mediate endoplasmic reticulum stress and immune reprogramming for periodontitis therapy. *Adv. Sci. (Weinh)*10.1002/advs.202302029 (2023). 10.1002/advs.202302029 PMC10520618 37452425

[203] Charlier E (2019). Chondrocyte dedifferentiation and osteoarthritis (OA). Biochem. Pharm..

[204] Wang XD, Zhang JN, Gan YH, Zhou YH (2015). Current understanding of pathogenesis and treatment of TMJ osteoarthritis. J. Dent. Res..

[205] Zhang S (2019). MSC exosomes alleviate temporomandibular joint osteoarthritis by attenuating inflammation and restoring matrix homeostasis. Biomaterials.

[206] Luo P, Jiang C, Ji P, Wang M, Xu J (2019). Exosomes of stem cells from human exfoliated deciduous teeth as an anti-inflammatory agent in temporomandibular joint chondrocytes via miR-100-5p/mTOR. Stem Cell Res. Ther..

[207] Hu S (2023). Dental pulp stem cell-derived exosomes revitalize salivary gland epithelial cell function in NOD mice via the GPER-mediated cAMP/PKA/CREB signaling pathway. J. Transl. Med..

[208] Kim JM (2022). Tonsil mesenchymal stem cells-derived extracellular vesicles prevent submandibular gland dysfunction in ovariectomized rats. Aging (Albany NY).

[209] AbuBakr N, Haggag T, Sabry D, Salem ZA (2020). Functional and histological evaluation of bone marrow stem cell-derived exosomes therapy on the submandibular salivary gland of diabetic Albino rats through TGFβ/ Smad3 signaling pathway. Heliyon.

[210] Salem ZA, Kamel AHM, AbuBakr N (2021). Salivary exosomes as a new therapy to ameliorate diabetes mellitus and combat xerostomia and submandibular salivary glands dysfunction in diabetic rats. J. Mol. Histol..

[211] Xiao, X.-Y., Zhang, N.-N., Long, Y.-Z. & Huang, G.-L. Repair mechanism of radiation-induced salivary gland injury by hypoxia-pretreated human urine-derived stem cell exosomes. *Oral Dis.*10.1111/odi.14476 (2022). 10.1111/odi.14476 36546840

[212] Franco AC, Aveleira C, Cavadas C (2022). Skin senescence: mechanisms and impact on whole-body aging. Trends Mol. Med..

[213] Tigges J (2014). The hallmarks of fibroblast ageing. Mech. Ageing Dev..

[214] Kim S, Lee SK, Kim H, Kim TM. Exosomes secreted from induced pluripotent stem cell-derived mesenchymal stem cells accelerate skin cell proliferation. *Int. J. Mol. Sci.* **19**, 3119 (2018). 10.3390/ijms19103119 PMC6213597 30314356

[215] Xia W (2022). Young fibroblast-derived exosomal microRNA-125b transfers beneficial effects on aged cutaneous wound healing. J. Nanobiotechnology.

[216] Go YY, Lee CM, Ju WM, Chae S-W, Song J-J (2021). Extracellular vesicles (secretomes) from human trophoblasts promote the regeneration of skin fibroblasts. Int. J. Mol. Sci..

[217] Takeo M, Lee W, Ito M (2015). Wound healing and skin regeneration. Cold Spring Harb. Perspect. Med..

[218] Pulido T, Velarde MC, Alimirah F (2021). The senescence-associated secretory phenotype: fueling a wound that never heals. Mech. Ageing Dev..

[219] Mervis JS, Phillips TJ (2019). Pressure ulcers: pathophysiology, epidemiology, risk factors, and presentation. J. Am. Acad. Dermatol..

[220] Shearer SC (2021). Facial pressure injuries from prone positioning in the COVID-19 era. Laryngoscope.

[221] Chen B (2019). Human embryonic stem cell-derived exosomes promote pressure ulcer healing in aged mice by rejuvenating senescent endothelial cells. Stem Cell Res Ther..

[222] Khosla S, Farr JN, Tchkonia T, Kirkland JL (2020). The role of cellular senescence in ageing and endocrine disease. Nat. Rev. Endocrinol..

[223] Ko KI, Sculean A, Graves DT (2021). Diabetic wound healing in soft and hard oral tissues. Transl. Res..

[224] Li X (2018). Exosomes from adipose-derived stem cells overexpressing Nrf2 accelerate cutaneous wound healing by promoting vascularization in a diabetic foot ulcer rat model. Exp. Mol. Med..

[225] Shiekh PA, Singh A, Kumar A (2020). Exosome laden oxygen releasing antioxidant and antibacterial cryogel wound dressing OxOBand alleviate diabetic and infectious wound healing. Biomaterials.

[226] Wang B (2023). Human fetal mesenchymal stem cells secretome promotes scarless diabetic wound healing through heat-shock protein family. Bioeng. Transl. Med..

[227] Jin S (2023). Young exosome bio-nanoparticles restore aging-impaired tendon stem/progenitor cell function and reparative capacity. Adv. Mater..

[228] Fukushima KA (2019). Screening of hydrogel-based scaffolds for dental pulp regeneration—a systematic review. Arch. Oral. Biol..

[229] Wang S (2023). Fabrication of an exosome-loaded thermosensitive chitin-based hydrogel for dental pulp regeneration. J. Mater. Chem. B.

[230] Ivica A, Ghayor C, Zehnder M, Valdec S, Weber FE (2020). Pulp-derived exosomes in a fibrin-based regenerative root filling material. J. Clin. Med..

[231] Swanson WB (2020). Controlled release of odontogenic exosomes from a biodegradable vehicle mediates dentinogenesis as a novel biomimetic pulp capping therapy. J. Control Release.

[232] Shang F (2021). Advancing application of mesenchymal stem cell-based bone tissue regeneration. Bioact. Mater..

[233] Liu A (2021). Optimized BMSC-derived osteoinductive exosomes immobilized in hierarchical scaffold via lyophilization for bone repair through Bmpr2/Acvr2b competitive receptor-activated Smad pathway. Biomaterials.

[234] Wei F, Li M, Crawford R, Zhou Y, Xiao Y (2019). Exosome-integrated titanium oxide nanotubes for targeted bone regeneration. Acta Biomater..

[235] Danhier F (2012). PLGA-based nanoparticles: an overview of biomedical applications. J. Control Release.

[236] Swanson WB (2020). Scaffolds with controlled release of pro-mineralization exosomes to promote craniofacial bone healing without cell transplantation. Acta Biomater..

[237] Li W (2018). Tissue-engineered bone immobilized with human adipose stem cells-derived exosomes promotes bone regeneration. ACS Appl. Mater. Interfaces.

[238] Kang Y (2022). Exosome-functionalized magnesium-organic framework-based scaffolds with osteogenic, angiogenic and anti-inflammatory properties for accelerated bone regeneration. Bioact. Mater..

[239] Han S (2023). Programmed release of vascular endothelial growth factor and exosome from injectable chitosan nanofibrous microsphere-based PLGA-PEG-PLGA hydrogel for enhanced bone regeneration. Int. J. Biol. Macromol..

[240] Gu C (2021). Technological advances of 3D scaffold-based stem cell/exosome therapy in tissues and organs. Front. Cell Dev. Biol..

[241] Sun X (2023). Mesenchymal stem cell-derived exosomes enhance 3D-printed scaffold functions and promote alveolar bone defect repair by enhancing angiogenesis. J. Pers. Med..

[242] Liu L (2023). Lithium-containing biomaterials stimulate cartilage repair through bone marrow stromal cells-derived exosomal miR-455-3p and histone H3 acetylation. Adv. Health. Mater..

[243] Zhang Z (2023). Enhancement of the therapeutic efficacy of mesenchymal stem cell-derived exosomes in osteoarthritis. Cell Mol. Biol. Lett..

[244] Witwer KW (2021). Updating MISEV: evolving the minimal requirements for studies of extracellular vesicles. J. Extracell. Vesicles.

[245] Rezaie J, Feghhi M, Etemadi T (2022). A review on exosomes application in clinical trials: perspective, questions, and challenges. Cell Commun. Signal.

